# Profound alterations of the chromatin architecture at chromosome 11p15.5 in cells from Beckwith-Wiedemann and Silver-Russell syndromes patients

**DOI:** 10.1038/s41598-020-65082-1

**Published:** 2020-05-19

**Authors:** Davide Rovina, Marta La Vecchia, Alice Cortesi, Laura Fontana, Matthieu Pesant, Silvia Maitz, Silvia Tabano, Beatrice Bodega, Monica Miozzo, Silvia M. Sirchia

**Affiliations:** 1 Medical Genetics, Department of Health Sciences, Università degli Studi di Milano, 20142 Milano, Italy; 2 Genome Biology Unit, Istituto Nazionale di Genetica Molecolare “Romeo ed Enrica Invernizzi” (INGM), 20122 Milano, Italy; 3 Fondazione IRCCS Ca’ Granda Ospedale Maggiore Policlinico, 20122 Milano, Italy; 4 Medical Genetics, Department of Pathophysiology and Transplantation, Università degli Studi di Milano, 20122 Milano, Italy; 5 Clinical Pediatric, Genetics Unit, MBBM Foundation, San Gerardo di Monza, 20900 Monza, Italy

**Keywords:** Genetics, Epigenetics, Medical genetics

## Abstract

Beckwith-Wiedemann syndrome (BWS) and Silver-Russell syndrome (SRS) are imprinting-related disorders associated with genetic/epigenetic alterations of the 11p15.5 region, which harbours two clusters of imprinted genes (IGs). 11p15.5 IGs are regulated by the methylation status of imprinting control regions ICR1 and ICR2. 3D chromatin structure is thought to play a pivotal role in gene expression control; however, chromatin architecture models are still poorly defined in most cases, particularly for IGs. Our study aimed at elucidating 11p15.5 3D structure, via 3C and 3D FISH analyses of cell lines derived from healthy, BWS or SRS children. We found that, in healthy cells, *IGF2/H19* and *CDKN1C/KCNQ1OT1* domains fold in complex chromatin conformations, that facilitate the control of IGs mediated by distant enhancers. In patient-derived cell lines, we observed a profound impairment of such a chromatin architecture. Specifically, we identified a cross-talk between *IGF2/H19* and *CDKN1C/KCNQ1OT1* domains, consisting in *in cis*, monoallelic interactions, that are present in healthy cells but lost in patient cell lines: an inter-domain association that sees ICR2 move close to *IGF2* on one allele, and to *H19* on the other. Moreover, an intra-domain association within the *CDKN1C/KCNQ1OT1 locus* seems to be crucial for maintaining the 3D organization of the region.

## Introduction

Genomic imprinting is a finely tuned epigenetic process fundamental for mammalian development, whereby, through specific patterns of DNA methylation and chromatin modification, only one copy of an imprinted gene (IG) is expressed, according to its parental origin^1^. Various perturbations of epigenetic programming are associated with human diseases, including imprinting disorders^2^.

Beckwith-Wiedemann (BWS OMIM #130650) and Silver-Russell (SRS OMIM #180860) syndromes are rare imprinting disorders that exhibit opposite growth abnormalities. The syndromes are caused by genetic and/or epigenetic alterations of the chromosome 11p15.5 region and show genetic heterogeneity (epigenetic defects are summarised in Supplementary Fig. S1). The 11p15.5 imprinted region spans about 1 Mb and includes *IGF2/H19* and *CDKN1C/KCNQ1OT1*, two clusters of IGs that are regulated by the methylation status of two imprinting control regions, ICR1 and ICR2, respectively^3^ (*IGF2/H19* region 275 Kb, *CDKN1C/KCNQ1OT1* region 470 Kb). In normal individuals, the paternally derived ICR1 allele is methylated, while the maternal allele is unmethylated; at ICR2, the opposite methylation pattern occurs. *IGF2* and *KCNQ1OT1* are expressed by the paternal allele, whereas *H19* and *CDKN1C* are expressed by the maternal allele^4^ (Supplementary Fig. S1a).

BWS is associated with following pathogenetic mechanisms: hypomethylation at ICR2 (about 50% of cases) (Supplementary Fig. S1b); mosaic segmental paternal uniparental disomy (UPD), that reflects an altered methylation as the fine-tuned balance of imprinting is disturbed (about 20% of cases) (Supplementary Fig. S1c); *CDKN1C* mutations of the maternal allele (5% of cases); hypermethylation at ICR1 (5% of cases) (Supplementary Fig. S1d); and 11p15 chromosomal rearrangements (3–5% of cases). Changes of the methylation status can be primary events or associated with genomic rearrangements.

SRS is associated with: hypomethylation of ICR1 (40–60% of cases) (Supplementary Fig. S1e); maternal UPD of chromosome 7 (4–10% of cases); chromosome 7 deletions/duplications (rare); and duplication of maternal 11p15.5 (unknown frequency)^3^.

In BWS, these molecular alterations trigger over-expression of paternal chromosome IGs (*IGF2* and *KCNQ1OT1*) and/or defective expression of IGs from the maternal chromosome (*H19* and *CDKN1C*). In SRS, by contrast, they cause an excess of *H19* expressed from the maternal chromosome and defective expression of *IGF2* from the paternal allele^5^. Importantly, in the majority of cases of BWS and SRS, the molecular defect is a mosaic condition; that is, it is present only in a fraction of cells^2,3^.

In eukaryotes, 3D chromatin organisation has various functions in numerous aspects of genome regulation including maintenance of genome stability, chromosome transmission, DNA replication, and gene expression. Indeed, transcriptional regulation is strongly affected by chromatin folding, where looping interactions facilitate the long-range control mediated by distant regulatory elements, such as enhancers^6–9^. In particular, enhancer-promoter interactions are primarily restricted within topologically associating domains (TADs)^9–11^, in which chromosomes are partitioned at the sub-megabase scale^12–15^. The most important TAD architectural proteins are CTCF (CCCTC-binding factor) and cohesins^16–18^.

Chromatin structure at the human *IGF2/H19 locus* differs between maternal and paternal alleles, and these parent-specific structures are required for correct expression of the IGs within this domain.

The *IGF2/H19* domain contains binding sites for several trans-acting factors such as ZFP57, involved in the establishment and maintenance of DNA methylation in imprinting control centres, OCT4 and SOX2, participating in maintaining hypomethylation of the maternal allele^19,20^. Moreover, the *IGF2/H19 locus* harbours a number of CTCF-binding site clusters that function cooperatively to form chromatin loops. These structures bring the enhancer into spatial proximity with its target promoter^21^. In particular, the unmethylated ICR1 of the maternal allele allows CTCF binding and prevents the *IGF2* gene from accessing enhancer downstream of *H19*. By contrast, the methylated ICR1 of the paternal allele does not bind CTCF, enabling the *IGF2* promoter and the enhancer region to interact^22,23^. The effects of abnormal methylation at ICR1 on the underlying chromatin and long-range associations with neighbouring CTCF sites are poorly understood^24,25^; however, Nativio and collaborators^24^ proposed that, in ICR1-related syndromes, a switch from the maternal to paternal conformation may occur in BWS and *vice versa* in SRS. No comprehensive description of 3D chromatin conformation at the *CDKN1C/KCNQ1OT1 locus* has been reported to date.

In this study, we investigated the 3D chromatin organisation of the 11p15.5 imprinted region in cells from healthy individuals and from patients with BWS and SRS, and found that profound alterations in the chromatin architecture of the *IGF2/H19* and *CDKN1C/KCNQ1OT1* regions characterise both imprinting disorders. Interestingly, we identified a cross-talk between the *IGF2/H19* and *CDKN1C/KCNQ1OT1* domains, based on a higher order of chromatin fold comprising the entire 11p15.5 imprinted region, resulting in complex 3D structural deregulation in the BWS and SRS cells.

## Results

We sought to investigate in depth the chromatin structure of chromosome region 11p15.5 and its potential deregulation in the related imprinting diseases (BWS and SRS), focusing particularly on *cis* regulatory elements, including enhancers and CTCF-binding sites. To achieve this aim, we have chosen the chromatin conformation capture (3C) approach since this method is appropriate to investigate the structure of a defined genomic region. Furthermore, to visualise the chromatin interactions, 3D FISH (Fluorescence *In Situ* Hybridization) using probe pairs across the region interrogated by 3C was applied.

### Detailed genomic landscape of the chromosome 11p15.5 imprinted region

**Figure 1:**
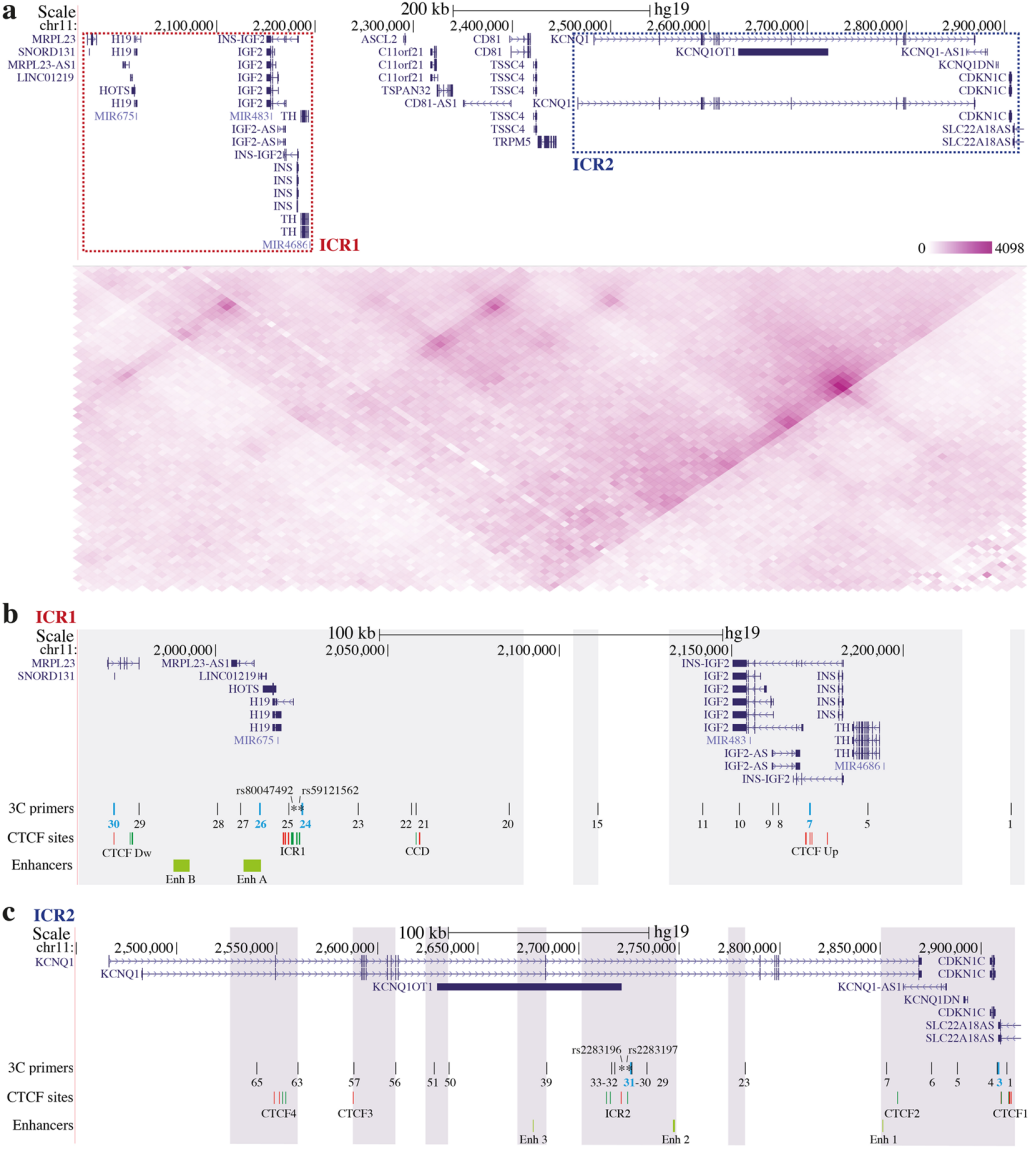
Schematic representation and Hi-C profile of the 11p15.5 *locus*. **(a**) The Figure is to scale. (top panel) UCSC Genome Browser map of the analysed region (from 1,960,000 to 2,920,000) showing the localisation of the genes. Red dashed rectangle marks ICR1 domain that includes *IGF2* and *H19* imprinted genes, while blue dashed rectangle highlights ICR2 domain that contains *CDKN1C* and *KCNQ1OT1* imprinted genes. (bottom panel) Hi-C profile of the 11p15.5 region, obtained using the WashU Epigenome Browser tool, showing the topologically associating domain (TAD) organisation (Resolution 5 kb). (**b,c**) Details of the genomic landscapes of the ICR1 (b; from 1,960,000 to 2,235,000) and ICR2 (c; from 2,450,000 to 2,920,000) *loci*. The locations of genes are indicated above. Analysed single nucleotide polymorphisms (SNPs; ‘rs’ numbers) are indicated by asterisks. The primers used for 3C experiments, corresponding to *Bgl*II restriction sites, are shown as vertical black lines, with anchor primers highlighted in light blue. Clusters of CTCF-binding sites (CTCF Up, CCD, ICR1, CTCF Dw, CTCF1/2/3/4, and ICR2) are indicated by red lines (reverse orientation) or green lines (forward orientation). Enhancer candidate regions (light green bars) are indicated below. 3C coverage is highlighted with grey areas.

The detailed landscape of the *IGF2/H19* and *CDKN1C/KCNQ1OT1* regions subjected to 3C analysis is depicted Fig. 1, which reports both literature information and data obtained interrogating specific database. The region spans approximately 1 Mb (chr11:1,922,706–2,916,895; UCSC Genome Browser GRCh37/hg19; https://genome.ucsc.edu) and harbours two IG clusters: *IGF2/H19* and *CDKN1C/KCNQ1OT1* (Fig. 1a top panel). Since chromosomes are organised in TADs, and considering the linear proximity of the two IG clusters, we investigated whether the two clusters lie within the same TAD. The Hi-C contact matrix, obtained using WashU Epigenome Browser tool (http://epigenomegateway.wustl.edu/)^15^ derived from the lymphoblastoid cell line GM12878, revealed that both *IGF2/H19* and *CDKN1C/KCNQ1OT1* domains localised in the same TAD (Fig. 1a bottom panel).

Many studies demonstrate that the binding of CTCF proteins at specific sites is important for the development of intra- and inter-chromosomal contacts^16–18^. Accordingly, we searched the CTCF-binding sites present in the regions covered in our 3C experiments. Analysing the ENCODE datasets of the lymphoblastoid cell line GM12878 present on UCSC genome browser and the literature^21,26^, we identified many CTCF-binding sites in both *IGF2/H19* and *CDKN1C/KCNQ1OT1* regions. The Supplementary Table S1 summarises the CTCF-binding sites, their genomic position, orientation and references. In particular, we identified four clusters of CTCF-binding sites in the *IGF2/H19* domain: one upstream of *IGF2* (CTCF Up), one downstream of *H19* (CTCF Down), one in the centrally conserved domain (CCD) region, and one in ICR1 (Fig. 1b). These clusters include the CTCF binding sites previously analysed in the studies about chromatin structure of the human *IGF2/H19 locus*^20,21,24,25^. In the *CDKN1C/KCNQ1OT1* region, five clusters of CTCF-binding sites were identified: one upstream of *CDKN1C* (CTCF1), one at the 3′ of *KCNQ1* (CTCF2), one close to ICR2 and two in the *KCNQ1* gene (CTCF3 and 4) (Fig. 1c, Supplementary Table S1).

Since the regulation of IGs involves numerous different mechanisms and *cis*-acting regulatory elements, such as enhancers, we searched for enhancer candidate regions, matching data published in the literature and the publicly available chromatin segmentation data from GM12878 cells, obtained *via* the Roadmap Epigenomics Project (ChromHMM software, http://compbio.mit.edu/ChromHMM/). For the *IGF2/H19* region, many studies suggest the involvement of shared enhancers regulating both *IGF2* and *H19* expression. In particular, two different regions that can act as enhancers have been identified (Fig. 1b, Enh A and Enh B)^27–31^. The *CDKN1C/KCNQ1OT1* domain contained three different enhancers candidate regions located in the 3′ of the *KCNQ1* gene (Enh 1), upstream of ICR2 (Enh 2), and in the middle of *KCNQ1OT1* (Enh 3) (Fig. 1c).

### Chromatin interactions at the 11p15.5 imprinted domain in cells from healthy individuals

At imprinted regions, chromatin can differ between the maternal and paternal alleles, and looping structures, mediated by regional CTCF-binding sites, are required for the correct expression of IGs^32^. Using 3C assay, we studied physical contacts between genes and regulatory elements at the human *IGF2/H19* and *CDKN1C/KCNQ1OT1 loci*.

Firstly, we set up the 3C positive controls using BACs and fosmids containing *IGF2/H19* and *CDKN1C/KCNQ1OT1* domains (Supplementary Fig. S2a,b).

Overall, two independent 3C experiments were performed for each sample, whose quality of 3C was checked as shown in Supplementary Fig. S2c,d.

To identify the main interactions within these *loci* in normal cells, we used two lymphoblastoid cell lines (CTRL1 and 2) generated from healthy children, showing normal methylation levels at both ICR1 and ICR2, by pyrosequencing analysis^33^ (Table 1). We reported the specific contacts between CTCF-binding sites, regulatory elements and genes mapping to *IGF2/H19* and *CDKN1C/KCNQ1OT1 loci* and defined these associations as primary interactions (Regions Of Interaction, ROIs).

**Table 1:** ICR1 and ICR2 methylation levels determined in peripheral blood lymphocytes and lymphoblastoid cell lines.

	Peripheral blood lymphocyte methylation level		Lymphoblastoid cell line methylation level	
	ICR1	ICR2	ICR1	ICR2
CTRL1	NA	NA	42%	43%
CTRL2	NA	NA	46%	44%
BWS-ICR1	58%	44%	78%	42%
BWS-ICR2	45%	15%	44%	16%
BWS-UPD	62%	34%	60%	27%
SRS-ICR1	28%	50%	28%	50%

ICR1 and ICR2 methylation levels, obtained by pyrosequencing, in peripheral blood lymphocytes and lymphoblastoid cell lines of controls and BWS and SRS patients.

Normal range: ICR1 40-52%, ICR2 39-50%^33^.

NA: not available.

Our results represent the superimposition of all frequent conformation states of these regions; moreover, 3C analysis revealed the sum of data from the two parental alleles.

#### Chromatin architecture analysis of the IGF2/H19 domain in control cell lines

**Figure 2:**
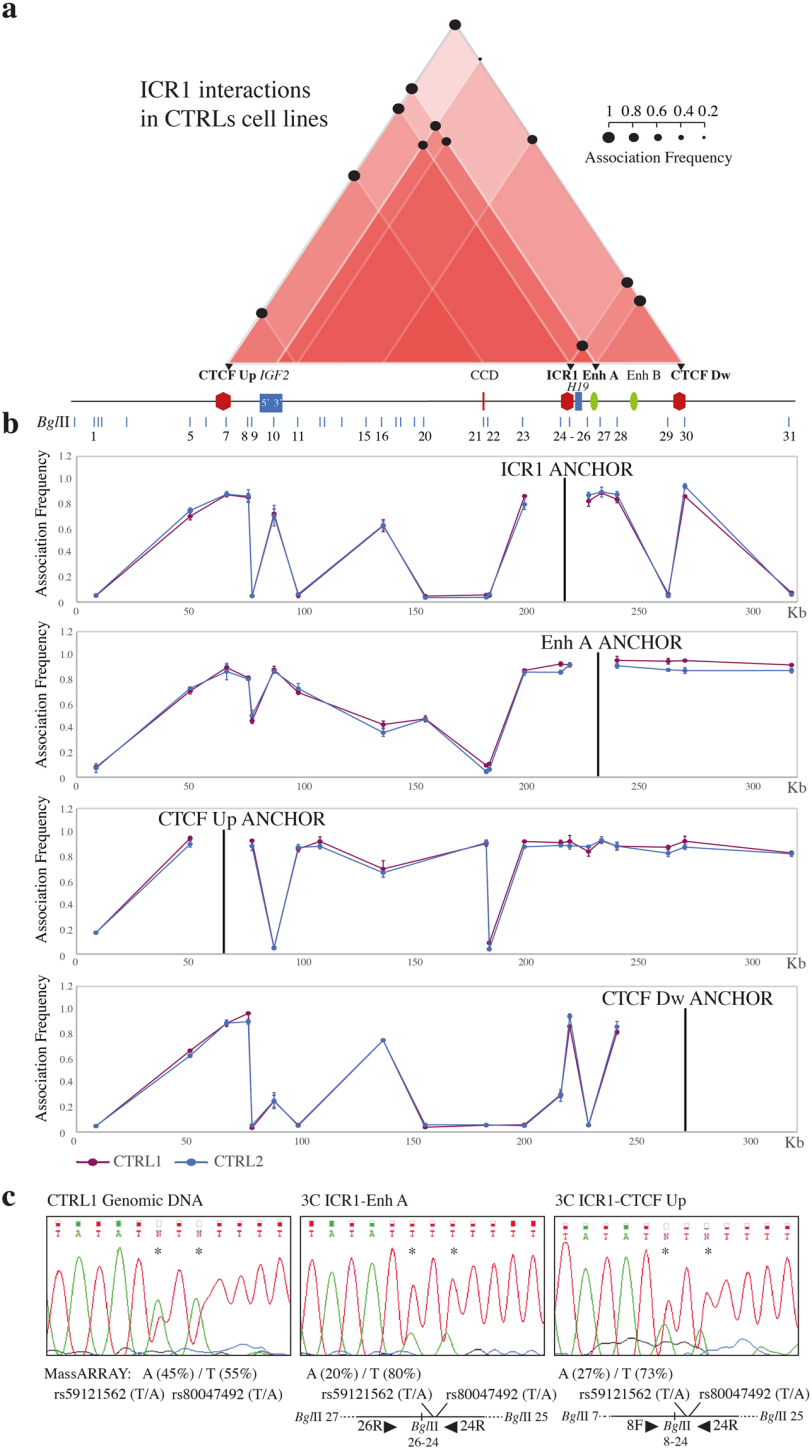
Chromatin interactions at the ICR1 domain determined by 3C experiments using control cell lines. The Figures a-b are to scale and have a reverse orientation with respect to Fig. 1 because these *loci* are classically reported with a reverse orientation since the genes are transcribed on the negative strand. (**a**) Schemes of the interactome for the ICR1 domain, showing the main ROIs observed in the control cell lines. Red triangles, interactions between different elements in the region; the intensity of the red colour is directly proportional to the number of interactions present in that sub-region. Black circles, mean association frequencies of the controls (CTRL1 and 2). A linear representation of the ICR1 imprinted domain is depicted below the interactome. Black triangles and bold characters indicate the anchors used for 3C analysis. The data of each control derive from two independent 3C experiments. The overall results represent the sum of the chromatin conformations of the paternal and maternal alleles. (**b**) ICR1 domain looping profiles for the different anchors in control cell lines CTRL1 (purple) and CTRL2 (blue). *Bgl*II restriction sites are indicated above. Each point in the profiles is the mean ± standard deviation of the two replicates for each one of the controls and indicates the association frequency between the anchor and the fragment on the left of the corresponding *Bgl*II restriction site. (**c**) Allele specificity of the ICR1-Enh A and ICR1-CTCF Up chromatin associations, in the heterozygous CTRL1 cell line, by 3C-SNP analysis. Qualitative and quantitative analyses of the two SNPs rs59121562 T/A and rs80047492 T/A (indicated by asterisks) were performed by Sanger sequencing and nucleic acid MALDI-TOF mass spectrometry (MassARRAY), respectively, and showed that the interactions were predominantly monoallelic (T and T alleles). The 3C ligation product with the orientation of primers (black triangles), SNPs, and *Bgl*II restriction sites is depicted below.

To study *IGF2/H19* region, we used four anchors: CTCF Up, ICR1, Enh A, and CTCF Dw (Fig. 1b). The 3C coverage is illustrated in Fig. 1b. The ROIs are summarised in the interactome scheme presented in Fig. 2a, and the details for each anchor are provided in Fig. 2b.

We noticed strong ROIs between the CTCF-binding sites; ICR1 interacted with CTCF Up and Dw, and CTCF Up also associated with CTCF Dw and CCD. In addition, Enh A interacted with ICR1, CTCF Up, and CTCF Dw. Interestingly, we also observed contacts engaging the regions at the 5′ and 3′ of *IGF2* in interactions with ICR1(5′ and 3′), Enh A (5′), and CTCF Up (3′) (Fig. 2a).

Consistent with previous studies^21,24^, our results confirm interactions that bring Enh A into spatial proximity with the *H19* and *IGF2* promoters. In addition, our data indicate that, also the Enh B, being close to the Enh A, is dragged near to the same promoters, suggesting a possible role of this enhancer in the IGs regulation.

About the human *IGF2/H19 locus*, models of parental allele-specific chromatin conformations were previously described. These reports allocated the associations CTCF Up-Enh A and CTCF Up-CTCF Dw to the paternal allele, and ICR1-CTCF Dw to the maternal allele. The interactions between CTCF Up-CCD and ICR1-CTCF Up were characterised as biallelic^21,24,25^. We could verify the allele specificity of the ICR1-CTCF Up and ICR1-Enh A associations, combining the 3C assay with SNPs analysis of the ligated products. This approach allows, in heterozygous samples, to distinguish whether the interactions involve one or both alleles. The analysis of the 3C products obtained from CTRL1 cell line, heterozygous for two SNPs (rs59121562 T/A and rs80047492 T/A) in the ICR1 restriction fragment, revealed that both interactions were predominantly monoallelic (TT haplotype, Fig. 2c).

To corroborate our 3C results, we performed virtual 4C from the lymphoblastoid cell line GM12878 using the tool available at http://promoter.bx.psu.edu/hi-c/virtual4c.php. As anchor points, we chosen the same used in 3C experiments of the *IGF2/H19 locus*. In particular, to better compare the data, we overlaid each 3C looping profile with the corresponding virtual 4C plot, aligning them on the x-axis. This analysis highlighted that the main interactions found using 3C approach were also visible in the 4C plots (Supplementary Figure S3).

Based on our and previous data we revised the models of the allele-specific architectural and functional loops at the *IGF2/H19* domain. The new maternal and paternal hypothetical models are presented in Supplementary Fig. S4.

#### Chromatin architecture analysis of the CDKN1C/KCNQ1OT1 domain in control cell lines

**Figure 3:**
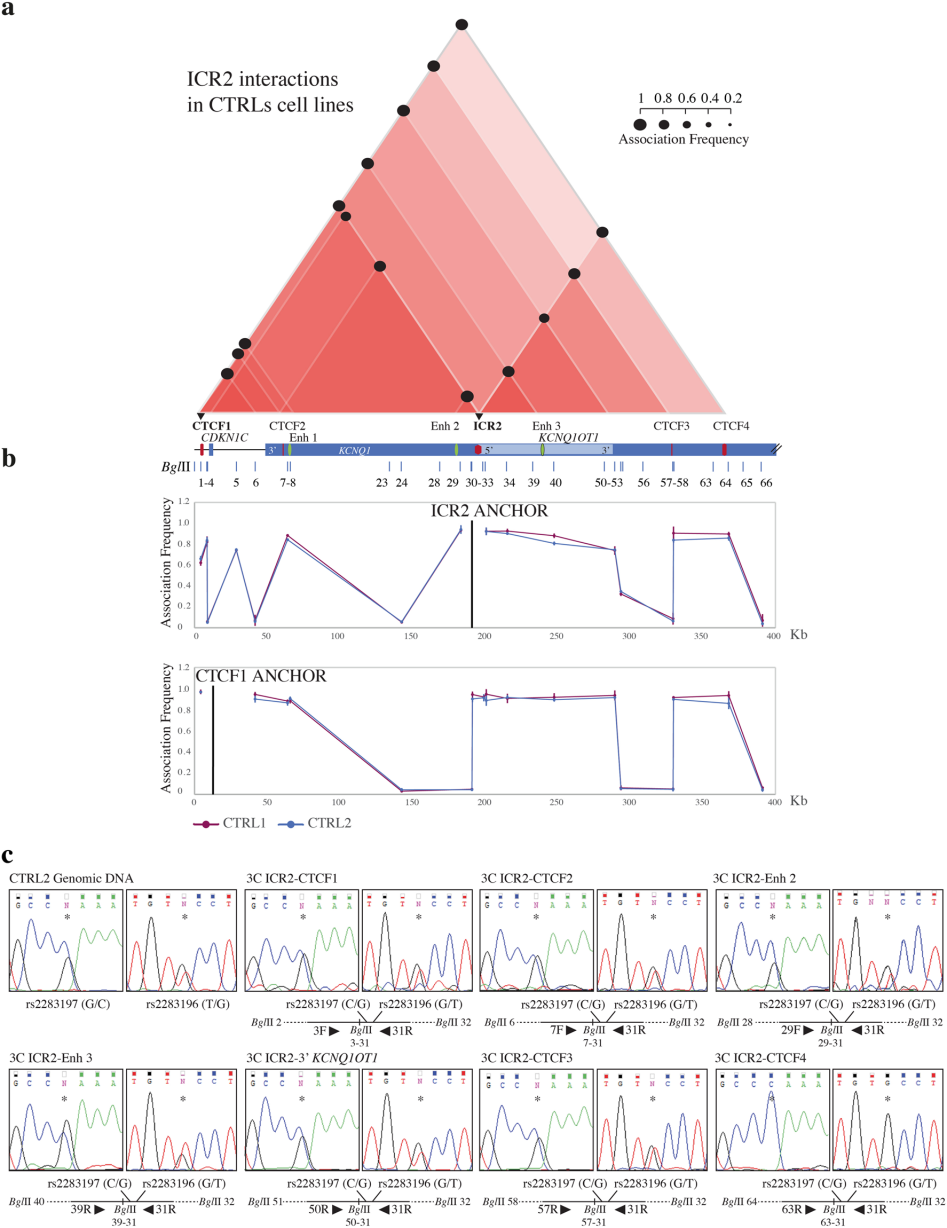
Chromatin interactions at the ICR2 domain determined by 3C experiments using control cell lines. The Figures a-b are to scale and have a reverse orientation with respect to Fig. 1 because these *loci* are classically reported with a reverse orientation since the genes are transcribed on the negative strand. (**a**) Schemes of the interactome for the ICR2 domain, showing the main ROIs observed in the control cell lines. Red triangles, interactions between different elements in the region; the intensity of the red colour is directly proportional to the number of interactions present in that sub-region. Black circles, mean association frequencies of the controls (CTRL1 and 2). A linear representation of the ICR2 imprinted domain is depicted below the interactome. Black triangles and bold characters indicate the anchors used for 3C analysis. The data of each control derive from two independent 3C experiments. The overall results represent the sum of the chromatin conformations of the paternal and maternal alleles. (**b**) ICR2 domain looping profiles for the different anchors in control cell lines CTRL1 (purple) and CTRL2 (blue). *Bgl*II restriction sites are indicated above. Each point in the profiles is the mean ± standard deviation of the two replicates for each one of the controls and indicates the association frequency between the anchor and the fragment on the left of the corresponding *Bgl*II restriction site. (**c**) Allele specificity of the ICR2 chromatin associations, in the heterozygous CTRL2 cell line, by 3C-SNP analysis. Analysis of the two SNPs rs2283197 G/C and rs2283196 T/G (indicated by asterisks) was performed by Sanger sequencing and showed that all interactions were biallelic, with the exception of CTCF4-ICR2, which was monoallelic (C and G alleles). The 3C ligation product, indicating the orientation of primers (black triangles), SNPs, and *Bgl*II restriction sites, is depicted below the electropherograms.

Differently from *IGF2/H19 locus*, the chromatin conformation at the *CDKN1C/KCNQ1OT1* domain has not been previously reported. We analysed this region using two anchors: ICR2 and the CTCF-binding sites upstream of the *CDKN1C* gene (CTCF1) (Fig. 1c). The 3C coverage is shown in Fig. 1c. The ROIs are summarised in Fig. 3a, and the details of the two anchors are presented in Fig. 3b.

We observed strong interactions between ICR2 and the CTCF-binding sites (CTCF1–4) within the domain. ICR2 also associated with two enhancers (Enh 2 and 3) within the *KCNQ1* gene. Both CTCF1 and ICR2 interacted with CTCF2–4, Enh 1, and Enh 3. Also in this domain, we found that the 5′ and 3′ regions of the IGs (5′ *CDKN1C*, 3′ *KCNQ1OT1*, and 3′ *KCNQ1*) associated with the CTCF-binding sites, CTCF1 and ICR2 (Fig. 3a). These data show that there are interactions between the regional enhancers (Enh 1–3) and IGs (5′ *KCNQ1OT1* and 5′ *CDKN1C*).

To assign allele specificity to the main ICR2 interactions, we performed 3C-SNP analysis in the control CTRL2 cell line, which resulted heterozygous at two SNPs (rs2283197 G/C and rs2283196 T/G) in the ICR2 restriction fragment (Fig. 3c). We found that only the ICR2-CTCF4 interaction was monoallelic (CG haplotype), indicating that CTCF4 binds the ICR2 predominantly on one allele, while all the others were biallelics (ICR2 and CTCF1, CTCF2, CTCF3, 3′ *KCNQ1OT1*, Enh 2, and Enh 3; Fig. 3c) and therefore occurring on both alleles.

As for the *IGF2/H19 locus*, we overlaid our 3C looping profiles with the virtual 4C plots of the *CDKN1C/KCNQ1OT1 locus* and confirmed the main 3C interactions (Supplementary Figure S5).

Also for *CDKN1C/KCNQ1OT1* domain, we devised possible models of allele-specific architectural and functional loops (Supplementary Fig. S6). These structures bring the regional enhancers (Enh 1–3) into the proximity of 5′ *KCNQ1OT1* in the paternal allele and of 5′ *CDKN1C* in the maternal allele.

In the two control cell lines, the interaction profiles of the *IGF2/H19* and *CDKN1C/KCNQ1OT1* domains overlapped completely, indeed, minimal standard deviations were observed (Figs. 2b and 3b). This suggests that the chromatin interactome identified in the two unrelated controls is highly specific and reproducible.

### Alteration of chromatin architecture at the *IGF2/H19* and *CDKN1C/KCNQ1OT1 loci* in cells from BWS and SRS patients

Changes in ICR1 and/or ICR2 methylation levels are associated with BWS and SRS and could cause alterations of chromosome 11p15.5 chromatin architecture. To explore the effects of abnormal methylation changes at the ICRs on chromatin conformation, we used 3C to analyse a panel of four lymphoblastoid cell lines derived from BWS and SRS patients with different epigenetic defects: SRS-ICR1 cell line with ICR1 hypomethylation (loss of ICR1 methylation on the paternal allele); BWS-ICR1 cell line with ICR1 hypermethylation (gain of ICR1 methylation on the maternal allele); BWS-ICR2 cell line with ICR2 hypomethylation (loss of ICR2 methylation on the maternal allele); BWS-UPD with methylation defects in both ICR1 and ICR2 due to paternal UPD 11p15.5 (double paternal contribution) (Supplementary Fig. S1). The specific methylation levels of the cell lines are reported in Table 1. We verified, in each patient, that the procedure of immortalization did not affect the methylation levels at the ICRs, comparing the methylation levels in DNAs from fresh peripheral blood lymphocytes and from lymphoblastoid cell lines after immortalization (Table 1). These data are in keeping with previous evidences showing that the methylation of the imprinting control regions remains very stable in culture^4,34,35^.

**Figure 4:**
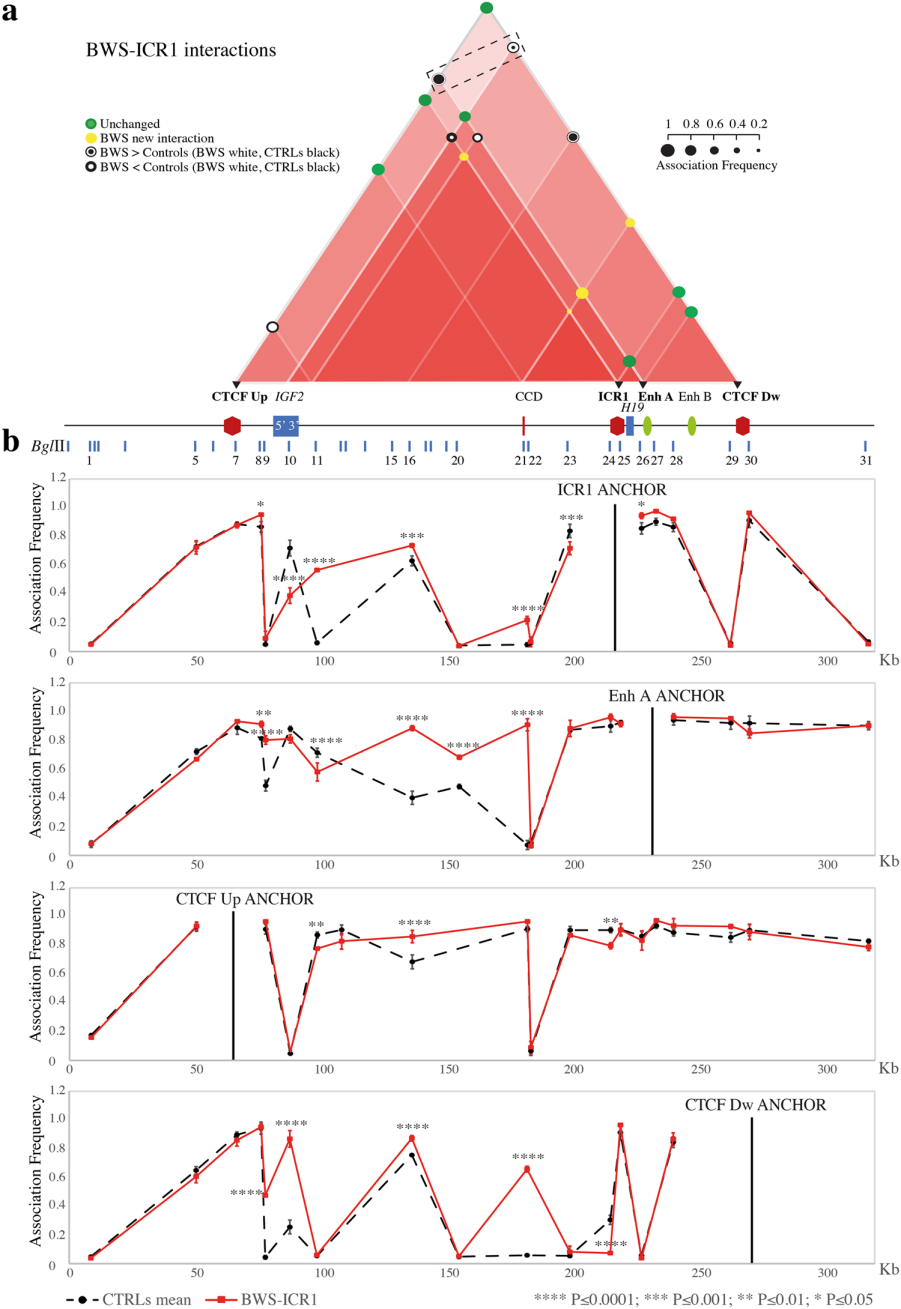
Chromatin architecture alterations at the ICR1 *locus* in BWS-ICR1 cells. The entire figure is to scale and has a reverse orientation with respect to Fig. 1 because these *loci* are classically reported with a reverse orientation since the genes are transcribed on the negative strand. (**a**) Schematic showing modifications of the chromatin interactome of the ICR1 domain in the BWS-ICR1 cell line compared with the mean of the controls. All the differences presented were statistically significant. Red triangles, interactions between different elements in the region. The intensity of the red colour is directly proportional to the number of interactions present in that sub-region. Coloured circles, association frequencies: green, unchanged interaction compared with the control mean; yellow, novel interaction in the pathological cell line; white circle >black circle, increase of the interaction strength in the pathological cell line compared with the control mean; black circle >white circle, decrease of the interaction strength in the pathological cell line compared with the control mean. A linear representation of the ICR1 imprinted domain is depicted below. Black triangles and bold characters indicate the anchor, used for 3C analysis. The dotted rectangle highlights the increased ROIs that indicate a gain of contact between enhancers (Enh A and Enh B) and *IGF2* promoter in the BWS-ICR1 cells. (**b**) The ICR1 *locus* looping profiles for the different anchors in controls (dotted black) and BWS-ICR1 (red) cell lines. *Bgl*II restriction sites are indicated above. Each point in the profile is the mean ± standard deviation of two independent 3C experiments and indicates the association frequency between the anchor and the fragment on the left of the corresponding *Bgl*II restriction site. Statistically significant differences (two-way ANOVA test) between the mean of controls and BWS-ICR1 are indicated by asterisks; ****P ≤ 0.0001; ***P ≤ 0.001; **P ≤ 0.01; *P ≤ 0.05. The overall results represent the sum of the chromatin conformations of normal alleles (paternal and maternal) and BWS pathological allele.

**Figure 5:**
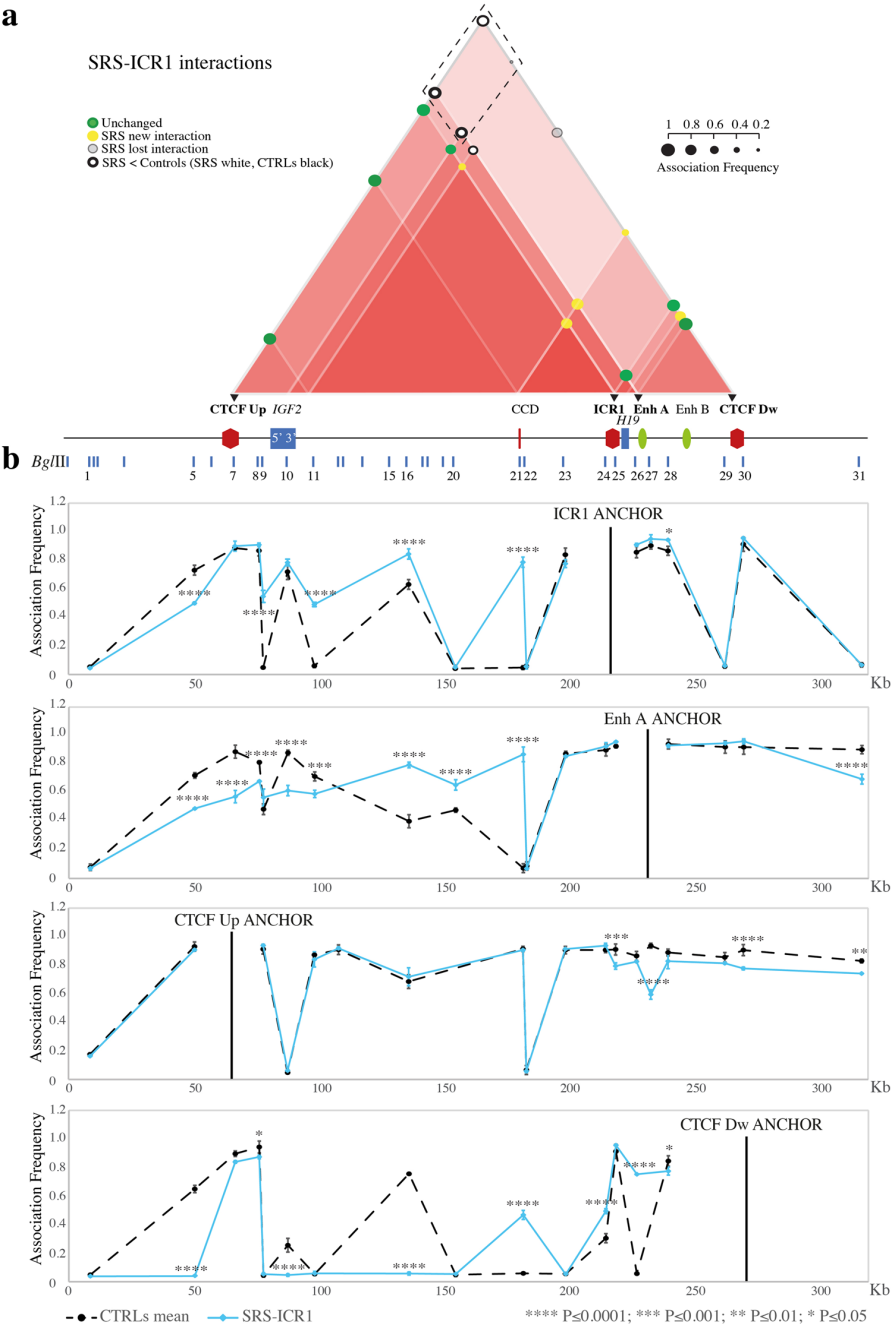
Chromatin architecture alterations at the ICR1 *locus* in SRS-ICR1 cells. The entire figure is to scale and has a reverse orientation with respect to Fig. 1 because these *loci* are classically reported with a reverse orientation since the genes are transcribed on the negative strand. (**a**) Schematic showing modifications of the chromatin interactome in the ICR1 domain in the SRS-ICR1 cell line compared with the mean of controls. All the differences displayed were statistically significant. Red triangles, interactions between the different elements in the region. The intensity of the red colour is directly proportional to the number of interactions present in a sub-region. Coloured circles represent association frequencies: green, unchanged interaction compared with the control mean; yellow, novel interaction in the pathological cell line; light grey, interaction lost in the pathological cell line; black circle >white circle, decrease of the interaction strength in the pathological cell line compared with the control mean. A linear representation of the ICR1 imprinted domain is depicted below. Black triangles and bold characters indicate the anchors, used for 3C analysis. The dotted rectangle highlights the decreased or lost ROIs that indicate a loss of contact between enhancers (Enh A and Enh B) and *IGF2* promoter in the SRS-ICR1 cells. (**b**) ICR1 *locus* looping profile for the different anchors in the controls (dotted black) and SRS-ICR1 (light blue) cell lines. *Bgl*II restriction sites are indicated above. Each point in the profile represents the mean ± standard deviation of two independent 3C experiments and indicates the association frequency between the anchor and the fragment to the left of the corresponding *Bgl*II restriction site. Statistically significant differences (two-way ANOVA test) between the mean of controls and SRS are indicated by asterisks; ****P ≤ 0.0001; ***P ≤ 0.001; **P ≤ 0.01; *P ≤ 0.05. Overall, the results represent the sum of the chromatin conformations of normal alleles (paternal and maternal) and the SRS pathological allele.

**Figure 6:**
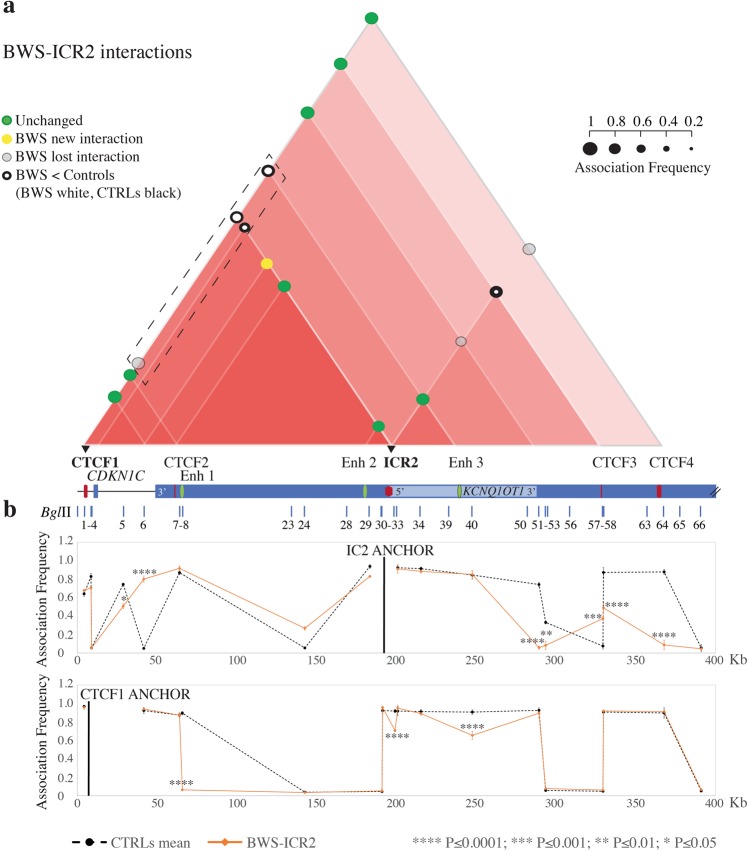
Chromatin architecture alterations at the ICR2 *locus* in BWS-ICR2 cells. The entire figure is to scale and has a reverse orientation with respect to Fig. 1 because these *loci* are classically reported with a reverse orientation since the genes are transcribed on the negative strand. (**a**) Schematic showing modifications in the chromatin interactome of the ICR2 domain in the BWS-ICR2 cell line compared with the mean of controls. All the differences displayed were statistically significant. Red triangles, interactions between different elements in the region. The intensity of the red colour is directly proportional to the number of interactions present in a sub-region. Coloured circles represent the association frequencies: green, unchanged interaction compared with the mean of controls; yellow, novel interaction in the pathological cell line; light grey, lost interaction in the pathological cell line; black circle> white circle, decrease in the interaction strength in the pathological cell line compared with the mean of controls. A linear representation of the ICR2 imprinted domain is depicted below. Black triangles and bold characters indicate the anchors, used for 3C analysis. The dotted rectangle highlights the decreased or lost ROIs that indicate a loss of contact between enhancers (Enh 1–3) and *CDKN1C* in the BWS-ICR2 cells. (**b**) The ICR2 *locus* looping profiles for the ICR2 and CTCF1 anchors in controls (dotted black) and BWS-ICR2 (orange) cell lines. *Bgl*II restriction sites are indicated above. Each point in the profile represents the mean ± standard deviation of two independent 3C experiments and indicates the association frequency between the anchor and the fragment to the left of the corresponding *Bgl*II restriction site. Statistically significant differences (two-way ANOVA test) between the mean of controls and BWS-ICR2 are indicated by asterisks: ****P ≤ 0.0001; ***P ≤ 0.001; **P ≤ 0.01; *P ≤ 0.05. The overall results represent the sum of the chromatin conformations of the normal alleles (paternal and maternal) and the BWS pathological allele.

In all the pathological cell lines we identified profound modifications in the chromosome 11p15.5 chromatin interactome, with changes in the strength or losses of numerous ROIs (Fig. 4, Fig. 5, Fig. 6 and Supplementary Fig. S7).

#### Chromatin architecture analysis of the IGF2/H19 domain in BWS-ICR1 and SRS-ICR1 cell lines

Analysis of samples from patients with opposite methylation defects at ICR1 (BWS-ICR1 and SRS-ICR1) revealed opposite variations in the *IGF2/H19* domain (Fig. 4 and Fig. 5). In BWS-ICR1, we observed an increase in the strength of the interactions among the regions upstream of *IGF2* and Enh A and between CTCF Dw and 5′ *IGF2*. In addition, we observed a decrease in the contacts between ICR1 and 5′ *IGF2* and between CTCF Up and 3′ *IGF2* (Fig. 4). By contrast, in SRS-ICR1 cells, we found a reduction in the strength of the interaction between Enh A and the *IGF2* region (CTCF Up, 5′ *IGF2*, and 3′ *IGF2*), together with a loss of contact between *IGF2* and CTCF Dw (Fig. 5). These results indicate that the ROIs that drag the enhancers (Enh A and Enh B) into proximity with the *IGF2* promoter (CTCF Up and 5′ *IGF2*) increase in the BWS-ICR1 cell line (Fig. 4a, dotted rectangle) and decrease in the SRS-ICR1 cell line (Fig. 5a, dotted rectangle), consistent with the upregulation of *IGF2* expected in BWS, and downregulation of *IGF2* expected in SRS. We did not observe any statistically significant change of the interaction between Enh A and *H19*, probably due to a proximity effect since the two regions are very close.

Interestingly, new interactions were also observed in the patient cell lines (BWS-ICR1 and SRS-ICR1), particularly between the CCD and *H19* regions and 3′ *IGF2* and ICR1 (Fig. 4 and Fig. 5).

#### Chromatin architecture analysis of the CDKN1C/KCNQ1OT1 domain in BWS-ICR2 cell line

Regarding the cell line with the ICR2 methylation defect (BWS-ICR2), we found variations in strength or complete loss of several ROIs in the *CDKN1C/KCNQ1OT1* domain (Fig. 6). In particular, we observed a loss of interaction between ICR2 and the downstream region (3′ *KCNQ1OT1*, CTCF3, and CTCF4), and a decrease in the associations among CTCF1 and ICR2/Enh 3 and between ICR2 and 5′ *CDKN1C*. Finally, we observed loss of the contact between CTCF1 and Enh 1, along with a new interaction of ICR2 and 3′ *KCNQ1* (Fig. 6). These data indicate that, in BWS-ICR2 cells, there is a reduction in the contacts between the regional enhancers (Enh 1–3) and *CDKN1C* (Fig. 6a, dotted rectangle), consistent with the expected downregulation of this gene in BWS with ICR2 hypomethylation.

In the BWS-ICR2 cell line the monoallelic ICR2-CTCF4 interaction was lost (Fig. 6a). We presume that the interaction is on the maternally derived allele, because the altered allele in BWS patients with ICR2 hypomethylation is of maternal origin.

#### Chromatin architecture analysis of the IGF2/H19 and CDKN1C/KCNQ1OT1 domains in BWS-UPD cell line

The BWS-UPD cell line, which harbours methylation defects in both ICRs, due to UPD which leads to a double dosage of the paternal allele, displayed chromatin architecture alterations at both *IGF2/H19* and *CDKN1C/KCNQ1OT1* domains (Supplementary Fig. S7). These variations only partially overlapped those observed in the other BWS cell lines, probably because the molecular defect of BWS-UPD cells, involving both ICRs, is more complex compared to BWS-ICR1 (defect in ICR1 only) and BWS-ICR2 (defect in ICR2 only).

### Higher order chromatin structure between the *IGF2/H19* and *CDKN1C/KCNQ1OT1* domains in cells from healthy individuals and BWS and SRS patients

Given the linkage between the two regions and their localisation within the same TAD (Fig. 1a bottom panel), we investigated whether the two domains were engaged in long-range contacts, and if these contacts can be compromised in BWS and SRS.

#### Interaction study between the IGF2/H19 and CDKN1C/KCNQ1OT1 domains

**Figure 7:**
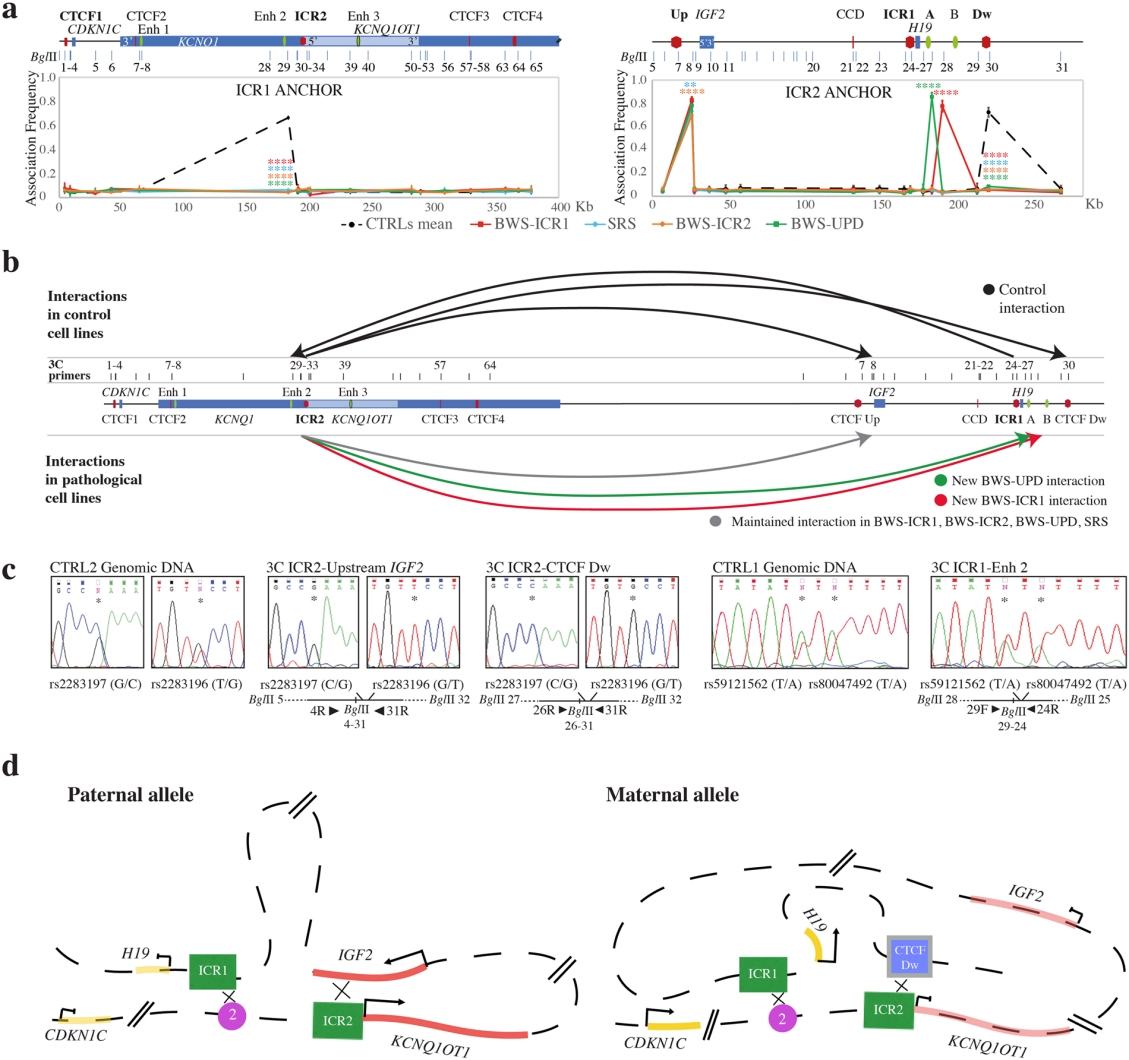
Higher order chromatin structure between the ICR1 and ICR2 domains in control and BWS/SRS cell lines. The figure has a reverse orientation with respect to Fig. 1. (**a**) ICR2 *locus* looping profile using ICR1 as the anchor (left), and ICR1 *locus* looping profile using ICR2 as the anchor (right) for controls and pathological cell lines. A schematic representation of the ICR1 (right) and ICR2 (left) domains, indicating the *Bgl*II restriction sites, is shown above. Each point in the looping profile represents the mean ± standard deviation of two independent 3C experiments for each cell line and indicates the association frequency between the anchor and the fragment to the left of the corresponding *Bgl*II restriction site. Statistically significant differences between the mean of the controls and pathological samples are indicated by asterisks (two-way ANOVA test; ****P ≤ 0.0001; ***P ≤ 0.001; **P ≤ 0.01; *P ≤ 0.05). (**b**) Sum of the interactions between the two imprinting domains, using ICR1 and ICR2 as anchors, in controls (top) and pathological cell lines (bottom). The genomic schematic represents the 11p15.5 region, CTCF-binding sites (red), enhancers (green), genes (blue), and 3C primers (black bars). (**c**) 3C-SNP analysis corresponding to the interactions in CTRLs. The allele specificity of the ICR2-region upstream of *IGF2* and the ICR2-CTCF Dw associations was assessed by Sanger sequencing of the SNPs rs2283197 G/C and rs2283196 T/G (indicated by asterisks) in CTRL2 informative cell line. The 3C-SNP analysis was also performed in CTRL1 informative cells for the ICR1-Enh 2 interaction by sequencing of the SNPs rs59121562 T/A and rs80047492 T/A (indicated by asterisks). The 3C ligation product, showing the orientation of primers (black triangles), SNPs, and *Bgl*II restriction sites, is depicted below the electropherograms. (**d**) Schematic representation of the parental-specific interactions between the two domains in CTRLs. Paternal: ICR1-Enh 2 and ICR2-region upstream *IGF2* contacts. Maternal: ICR1-Enh 2 and ICR2-CTCF Dw interactions. Regional elements: blue rectangle, CTCF-Dw binding site cluster; green rectangle, ICR1 and ICR2; violet circle, enhancer 2; red line, paternally expressed *IGF2* and *KCNQ1OT1*; yellow line, maternally expressed *H19* and *CDKN1C*.

We used ICR1 as the anchor for 3C analysis of the *CDKN1C/KCNQ1OT1* domain and ICR2 as the anchor for interrogate of the *IGF2/H19* region. In control cell lines, we identified some inter-associations between the two domains (Fig. 7a,b). ICR1 interacted with Enh 2, and ICR2 interacted with the region upstream of *IGF2* and with CTCF Dw. We evaluated the allele specificity of these interactions by performing 3C-SNP assays in the 3C products of the CTRL1 (heterozygous for SNPs in ICR1 restriction fragment as described above) to evaluate the ICR1 association, and in the 3C products of the CTRL2 (heterozygous for SNPs in ICR2 restriction fragment as described above) to assess ICR2 contacts. Interestingly, ICR2 monoallelically associated with the region upstream of *IGF2* (GT haplotype) and with the CTCF Dw (CG haplotype), whereas ICR1 biallelically interacted with Enh 2 (Fig. 7c). In CTRL2, therefore, the haplotypes at ICR2 are GT and CG, even if the parental origin is undetermined. However, based on the genotype, these results indicate that the two interactions engaging the ICR2 involve one allele only, one contact is on the paternal derived allele and the other on the maternal one.

We summarised the parental-specific interactions between the two domains in Fig. 7d.

In pathological conditions, the profile of these contacts was altered; we observed the complete loss of the ICR1-Enh 2 and ICR2-CTCF Dw inter-associations and the emergence of novel interactions (ICR2-Enh A in BWS-UPD and ICR2-Enh B in BWS-ICR1; Fig. 7a,b). In particular, the results obtained on BWS-ICR2 cell line showed that the ICR2-CTCF Dw monoallelic interaction was lost whereas the ICR2-Upstream IGF2 monoallelic association was maintained. Since the altered allele is of maternal origin in BWS with ICR2 hypomethylation, we think that ICR2-CTCF Dw association is likely on the altered maternal allele whereas the ICR2-Upstream IGF2 interaction is on the normal paternal allele. Based on these results we infer that the CG and GT haplotypes are on the maternal and paternal alleles, respectively.

#### Influence of the chromatin structure defects of one domain on the other

Given the inter-associations of the two imprinted regions, we tested if defects in one domain could influence the structure of the other one. We performed 3C analysis of the *IGF2/H19* domain in patient cell lines with driver methylation defects in ICR2 and of the *CDKN1C/KCNQ1OT1* domain in patient cell lines with driver methylation defects in ICR1. Surprisingly, we found alterations in the interaction profile of the *CDKN1C/KCNQ1OT1* region in the BWS-ICR1 and SRS-ICR1 cell lines and, similarly, of the *IGF2/H19* domain in BWS-ICR2 cells, compared to the CTRLs (Supplementary Fig. S8). These data indicate that a methylation defect at the *IGF2/H19 locus* can also affect the chromatin conformation of the *CDKN1C/KCNQ1OT1* domain and *vice versa*. In light of these observations, we think that this phenomenon could be caused by the loss of the inter-associations between the *IGF2/H19* and *CDKN1C/KCNQ1OT1 loci*, when one of two domains is architecturally altered, the normal inter-domain contacts become compromised, also triggering alteration of the other domain.

### Visualization of the higher order chromatin structure between the *IGF2/H19* and *CDKN1C/KCNQ1OT1* domains by 3D FISH

**Figure 8:**
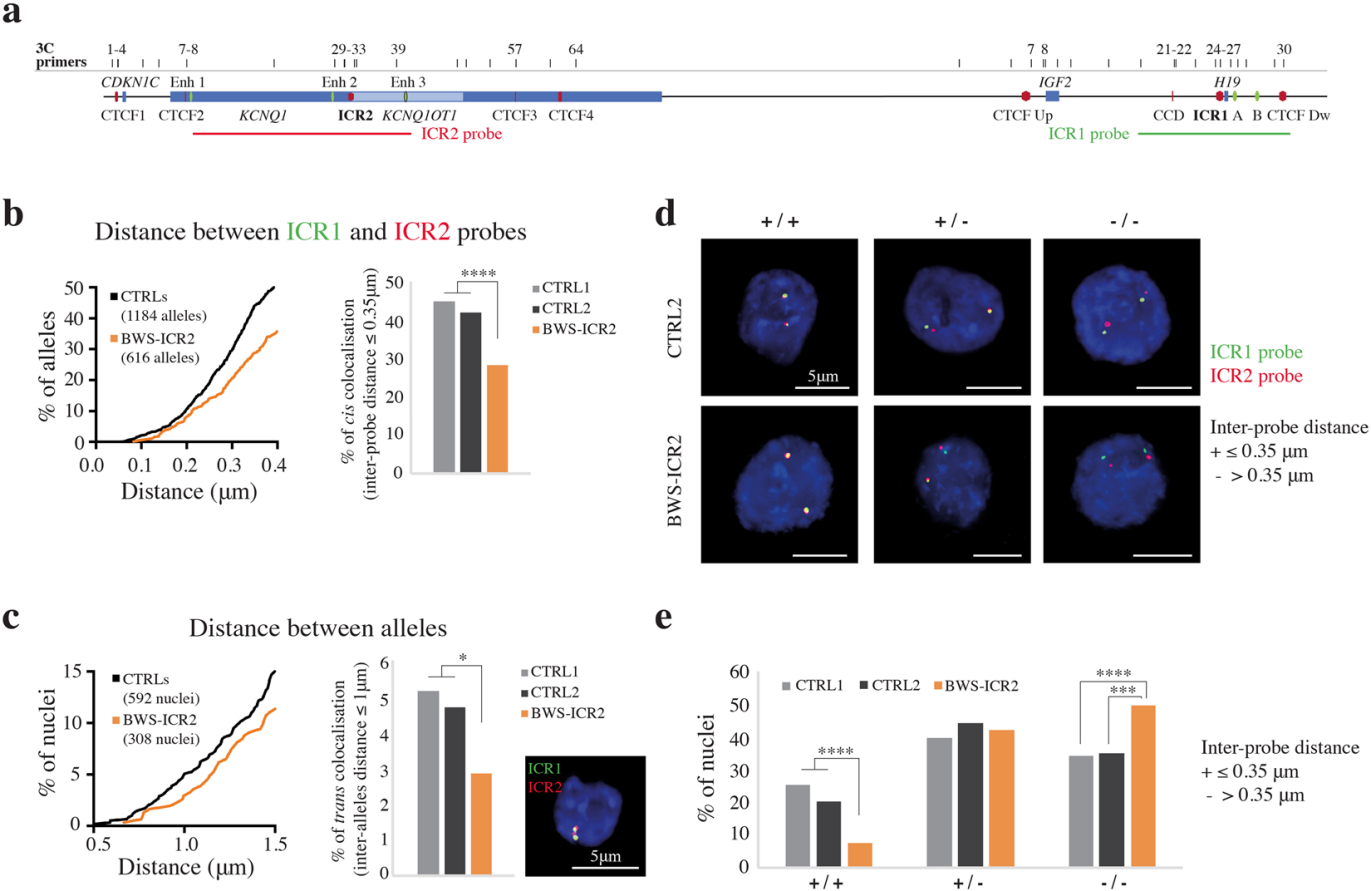
Visualization of the higher order chromatin structure between the *IGF2/H19* and *CDKN1C/KCNQ1OT1* domains by 3D FISH. (**a**) Schematic representation of the 11p15.5 region with a reverse orientation with respect to Fig. 1. CTCF-binding sites (red), enhancers (green), genes (blue), 3C primers (black bars) and 3D FISH probes (red line for ICR2 probe and green line for ICR1 probe). (**b–e**) Two-color 3D DNA FISH in CTRLs and BWS-ICR2 cell lines. Nuclei analysed: n = 302 (604 alleles) for CTRL1, n = 290 (580 alleles) for CTRL2 and n = 308 (616 alleles) for BWS-ICR2. 3D centroids distances (μm) were measured between ICR1 probe (green) and ICR2 probe (red) signals of each allele for the *cis*-interactions (0.35 μm cut-off), and between the doublet green-red signal of the two alleles for the *trans*-associations (1 μm cut-off). Nuclei are counterstained with DAPI (blue). (**b**) *Cis*-interactions evaluation (distance between ICR1 and ICR2 probes). Left: detail of cumulative frequency distribution plot showing the percentage of alleles at increasing inter-probe distances. Right: bar plot displaying the percentage of alleles with inter-probe distance ≤ 0.35 μm cut-off. Statistically significant differences between controls and BWS-ICR2 samples are indicated by asterisks (unpaired t-test; ****P ≤ 0.0001). (**c**) *Trans*-interactions evaluation (distance between alleles). Left: detail of cumulative frequency distribution plot showing the percentage of nuclei at increasing inter-doublet distances. Middle: bar plot displaying the percentage of nuclei with inter-doublet distance ≤1 μm cut-off. Statistically significant differences between controls and BWS-ICR2 samples are indicated by asterisks (unpaired t-test; *P ≤ 0.05). Right: representative example of nucleus with *trans*-interactions. (**d,e**) Pattern of ICR1 and ICR2 probes interactions in the two parental alleles in controls and BWS-ICR2 nuclei. (**d**) Example of nuclei classification: +/+ with ICR1-ICR2 probes distance ≤0.35 μm on both alleles (colocalised spots), +/− with colocalising probes on only one allele, −/− with inter-probe distance >0.35 μm on both alleles. (**e**) Bar plots of the percentage of nuclei for each category in CTRL1, CTRL2 and BWS-ICR2. Statistically significant differences between controls and BWS-ICR2 samples are indicated by asterisks (Fisher’s exact test; ****P ≤ 0.0001; ***P ≤ 0.001).

To validate the 3C results, we performed 3D DNA FISH experiments in controls and BWS-ICR2 cell lines. We chose to analyse the BWS-ICR2 cell line because it lost almost all the interactions between the two domains observed in 3C experiments and did not show new contacts (Fig. 7). Two probes, one spanning the region between CCD and CTCF Dw (ICR1-probe, green) and the other covering CTCF2-ICR2 region (ICR2-probe, red; Fig. 8a) were used. Each allele is marked by a doublet red-green signal. We measured the 3D centroids distances between the green and red signals for each allele to evidence the *cis*-interactions (intra-allele interaction), and between the green-red doublet of one allele and that of the other allele, to detect the *trans*-associations (inter-alleles interaction). For *cis*-interactions we considered the inter-probe FISH distance ≤ 0.35 µm (category representing paired signals) as indicative of colocalisation^36,37^. For *trans*-associations, a distance of 1 μm between the doublet of the two alleles was chosen as upper cut-off^38,39^.

#### Evaluation of the cis and trans-interactions in control and BWS-ICR2 cell lines

Figure 8b represents the distribution of the distances between the green and red probes for each allele in controls (CTRL1 302 nuclei, 604 alleles and CTRL2 290 nuclei, 580 alleles) and in BWS-ICR2 (308 nuclei, 616 alleles). In controls, we observed a high rate of *cis*-colocalisation, with a 44.06% of the alleles showing an inter-probe distance ≤ 0.35 µm (mean of the CTRLs). These data confirm the 3C results on the interactions between *IGF2/H19* and *CDKN1C/KCNQ1OT1* domains, clearly showing that the most frequently associations are *in cis*.

The frequency of *cis*-colocalisation was drastically reduced (28.73% of the alleles, P ≤ 0.0001) in BWS-ICR2 cell line (Fig. 8b, right), as expected by 3C results, that showed loss of ICR2-CTCF Dw and ICR1-Enh 2 interactions (Fig. 7a).

The monoallelic ICR2-Upstream *IGF2* association was still present in the BWS-ICR2 cells (Fig. 7a), and could explain the 28.73% of the *cis*-interactions observed in this cell line. This contact most likely is engaged on the normal paternal allele.

Interestingly, we found that the two parental alleles were also involved in *trans*-associations, since in the 5.06% of controls nuclei we observed the two doublets within 1 µm (Fig. 8c). In BWS-ICR2 the nuclei that displayed *trans*-interactions were significantly reduced (2.92%, P ≤ 0.05, Fig. 8c, middle).

#### Pattern of ICR1 and ICR2 probes interactions in the two parental alleles in control and BWS-ICR2 cell lines

To understand how the two parental alleles appeared in each nucleus, we classified the nuclei into three different categories (Fig. 8d, e): 1) +/+ or *cis*-interaction present in both alleles, when the red and green spots of each doublet were separated by a distance ≤ 0.35 μm (colocalised *loci*); 2) +/− when one allele showed *cis*-association (distance ≤ 0.35 μm) and the other did not (distance > 0.35 μm); 3) −/− or *cis*-interaction absent in both alleles (distance > 0.35 μm).

In controls, the 23% of nuclei were +/+ (25.5% CTRL1 and 20.34% CTRL2), the 42.23% +/− (40.07% CTRL1 and 44.5% CTRL2) and the 34.8% −/− (34.44% CTRL1 and 35.17% CTRL2) (Fig. 8e). In BWS-ICR2, the +/+ and −/− categories where significantly changed compared to controls, indeed only the 7.47% of nuclei displayed *cis*-associations on both alleles (P ≤ 0.0001), whereas the BWS-ICR2 nuclei without interactions increased up to 50% (P ≤ 0.001, Fig. 8e).

Overall, evidence from visual analyses corroborates the 3C results and indicates that in BWS-ICR2 cells one allele, purportedly the maternal one, is dysregulated due to the loss of both *cis*- and *trans*-interactions.

We did not observe significant difference of the +/− category. This may be because the loss of the interaction between the two domains of one allele in BWS-ICR2 cells cause the shift from +/+ to +/− and from +/− to −/− of some nuclei, maintaining unchanged the category +/−.

## Discussion

Knowledge of the 3D chromatin organisation at chromosome 11p15.5 may help to improve understanding of the pathomechanisms underlying BWS and SRS imprinted disorders, typically associated with 11p15.5 alterations. Here, we studied the chromatin conformation of the *IGF2/H19* and *CDKN1C/KCNQ1OT1* domains using 3C assays and 3D FISH in normal cell lines, to define the regional configuration of the physical interactions between the target genes and elements involved in gene regulation. Our data extend the available information regarding the structure of the *IGF2/H19* domain and define, for the first time, the interactome of the *CDKN1C/KCNQ1OT1* domain and the long-range contacts in which the two domains engage. We also evaluated the 3D architecture of these regions in cells from patients with BWS and SRS to assess the effects of genetic and/or epigenetic defects at 11p15.5 on regional chromatin architecture, and we identified profound alterations in the structure of the entire imprinted domain.

In cells from healthy controls, we found several interactions at the *IGF2/H19* and *CDKN1C/KCNQ1OT1 loci*, which appear to be stable and reveal a complex chromatin structure, reflecting the fine-tuning of parental allele-specific regulation of IGs. Most of these interactions were confirmed by the comparison between our 3C results and the virtual 4C data (Supplementary Figures S3 and S5). However, some interactions are more appreciable by 3C due to the different resolution of the two techniques, suggesting that the 3C approach is more suitable for the analysis of small regions.

We revised previous models^21,24^ of the allele-specific architectural loops at the *IGF2/H19* domain also in the light of our findings, hypothesizing new maternal and paternal models as reported in the Supplementary Fig. S4.

Very few data are available about chromatin interactions at the *CDKN1C/KCNQ1OT1* domain^40^, and no models of the 3D chromatin structure of this *locus* have been reported. We found that the interactome of this *locus* is characterised by several ROIs involving the CTCF clusters, enhancers and imprinted genes. We devised the possible parental-specific models of the ICR2 domain (see Supplementary Fig. S6 for details), containing different architectural and functional loops that drag the regional enhancers (Enh 1–3) near the expressed IGs (*CDKN1C* on the maternal allele and *KCNQ1OT1* on the paternal allele), while isolating the silenced alleles (paternal *CDKN1C* and maternal *KCNQ1OT1*).

This study included cell lines generated from patients with BWS and SRS, in which drastic perturbations of the interaction profiles of both *IGF2/H19* and *CDKN1C/KCNQ1OT1* domains were discovered. Interestingly, we identified syndrome-specific associations that recurred among the patient cell lines irrespective of their genetic/epigenetic defect, suggesting the existence of novel alleles with abnormal chromatin conformation. These data may indicate that, even if in BWS and SRS the methylation of an ICR shifts from maternal to paternal status or *vice versa*, this is not sufficient for the opposite chromatin conformation to be assumed. In Supplementary Fig. S9 we present a possible model of BWS-ICR1 and SRS-ICR1 pathological alleles, that takes into account the novel contacts and modifications in the interactome observed in the pathological cell lines. In these pathological alleles, the spatial repositioning of the regional enhancers, together with the methylation status of ICR1, could contribute to the IG expression defects that characterise BWS and SRS carrying ICR1 defects. Also, in the *CDKN1C/KCNQ1OT1* domain, the loss of interactions between *CDKN1C* and the regional enhancers observed in BWS-ICR2 is consistent with the altered expression of this gene observed in patients with BWS and ICR2 hypomethylation.

Interestingly, we identified a crucial monoallelic interaction between ICR2 and a CTCF-binding site within *KCNQ1* (CTCF4) that is lost in BWS-ICR2 cells. In this CTCF cluster Demars and collaborators^41^ previously reported genetic variants that affect CTCF binding and confer a risk of BWS on maternal transmission. Taken together these observations suggest a key role of ICR2-CTCF4 interaction in establishing the 3D structure of the region. Therefore, the loss of this interaction could lead to the alteration of the imprinted genes expression.

Overall, these data highlight the importance of long-range interactions in the control of IGs expression.

A significant finding of this study is the observation of unexpected alterations in the *IGF2/H19* domain interactome in a BWS cell line with a driving defect in ICR2 and *vice versa*. These results suggest that the conformation of one *locus* influences the 3D structure of the other. Also, SRS-ICR1 cells exhibited changes at the *CDKN1C/KCNQ1OT1* domain, supporting the idea of interconnection between the chromatin conformations of the two regions. By 3C and 3D FISH approaches on controls cells, we demonstrate the existence of inter-domain interactions that are lost or changed under pathological conditions. These data lead us to conclude that the 3D chromatin structure at 11p15.5 is more complex than expected, with different orders of chromatin organisation, from the level of the individual domains to a higher order structure between the two domains. These inter-domains contacts could be critical to the correct looping within the single domains: the maternal allele of the *IGF2/H19* domain could require the maternal allele of the *CDKN1C/KCNQ1OT1* domain to assume/maintain the proper chromatin conformation and *vice versa*, with the same phenomenon occurring at the paternal allele.

It is important to note that the inter-domain interactions between ICR2-region upstream of *IGF2* and ICR2-CTCF Dw (*H19*) are monoallelic (3C-SNP data) and are *cis*-interactions (3D FISH data), and they move *IGF2* close to ICR2 on one parental allele, and *H19* into proximity to ICR2 on the other allele. These data suggest the involvement of a mechanism to coordinate genes with the same expression status, even those distant from each other, as previously described for other genes and regions^42^ in this context, *IGF2* and *KCNQ1OT1* on the paternal allele and *H19* and *CDKN1C* on the maternal allele.

The 3C and 3D FISH results obtained in pathological cell lines highlight the alteration of the contacts between the two *loci*. These data could suggest significant rearrangements within the TAD, where the two control regions belong. The reconfiguration of the TAD could affect the expression of the imprinted genes and so have dramatic effect on the phenotype.

In this study we focused our attention on the role played by the interactions within and between *IGF2/H19* and *CDKN1C/KCN1OT1 loci* in the organisation of the 3D chromatin structure. Nevertheless, other regions beyond the 3C analysed range, could be involved in the chromatin architecture of the 11p15.5 imprinting domain.

In summary, we provide a refined characterisation of the chromatin architecture of the 11p15.5 imprinted domain and identified a cross-talk between the *IGF2/H19* and *CDKN1C/KCNQ1OT1* domains, based on *cis* inter-domains interactions, that was lost in BWS and in SRS; these inter-domain associations seem to be crucial to the formation/maintenance of the normal looping conformations within each domain. Moreover, we defined a monoallelic interaction between ICR2 with CTCF4 that seems to be crucial to establish the 3D structure of the *CDKN1C/KCNQ1OT1* region.

Based on these evidences, we also provided hypothetical models that attempt to explain the complex structure of 11p15.5 region and highlight regions with a key role in imprinting regulation and monoallelic expression, including those distant from ICRs, such as CTCF4.

## Methods

### Lymphoblastoid cell lines

Experiments were carried out using lymphoblastoid cell lines generated from two healthy children with normal ICR1 and ICR2 methylation (CTRL1 and CTRL2), three children patients with BWS, carrying different ICR1 and/or ICR2 methylation defects (ICR1 hypermethylation, BWS-ICR1; ICR2 hypomethylation, BWS-ICR2; and 11p15.5 pUPD, BWS-UPD), and one child with SRS with hypomethylation of ICR1 (SRS-ICR1) (Table 1).

Patients with BWS and SRS were clinically diagnosed following the clinical criteria for these conditions^3^. We carried out the molecular evaluation on peripheral blood lymphocytes from the patients that confirmed the clinical diagnosis (Table 1).

BWS and SRS patient lymphoblastoid cell lines were established from patient blood samples, by Epstein-Barr virus transformation at the Galliera Genetic Bank (a member of the Telethon Network of Genetic Biobanks; project no. GTB12001). Both normal and patient lymphoblastoid cell lines were cultured in RPMI 1640 medium supplemented with 10% foetal bovine serum (Euroclone) and antibiotics (antibiotic-antimycotic 100×, Euroclone) at 37 °C in 5% CO_2_.

### Ethics Statement

The study was approved by the Ethics Committee of Fondazione IRCSS Ca’ Granda Ospedale Maggiore Policlinico (no. 526/2015). Appropriate written informed consent was obtained from parents’ patients. All the procedures performed in this study were in accordance with the 1964 Helsinki declaration and its later amendments.

### DNA methylation analysis

Total DNA was extracted from lymphoblastoid cell lines using the QIAamp DNA Mini kit (Qiagen) according to the manufacturer’s instructions. Analyses of ICR1 and ICR2 methylation levels were performed as previously described^4,33,43^.

### Chromatin conformation capture assay

Chromatin conformation capture (3C) was performed as previously described with a few modifications^44–46^. In detail, 13 × 10^6^ lymphoblastoid cells were resuspended in 5 ml of crosslinking buffer (10 mM Tris pH 8, 50 mM NaCl, 10 mM MgCl_2_, 1 mM DTT, and protease inhibitor cocktail) and crosslinked with 2% formaldehyde for 10 min at room temperature. Reactions were quenched by the addition of glycine, and cells were subsequently washed with ice-cold PBS. Cells were then recovered and suspended in 10 ml of cold cell lysis buffer (10 mM Tris pH 8, 10 mM NaCl, 5 mM MgCl_2_, 0.1 mM EGTA, and protease inhibitor cocktail) and incubated on ice for 10 min. Lysis was completed with approximately twenty strokes using a Dounce homogenizer (Tight). Nuclei were collected by centrifugation and resuspended in 500 µl of 1.5× *Bgl*II restriction enzyme buffer (NEBuffer 3.1; New England Biolabs) with SDS to a final concentration of 0.3%, and samples were incubated at 37 °C for 1 h with gentle agitation. Triton X-100 was then added to a final concentration of 1.8% and incubated for a further 1 h at 37 °C. Digestion was performed overnight with 600 U of *Bgl*II at 37 °C with gentle agitation (see Fig. 1b, c for restriction map). The enzyme was then inactivated by the addition of SDS to a final concentration of 1.6% and incubation at 65 °C for 25 min. Reactions were diluted to a final digested chromatin concentration of 2.5 ng/µl in 1.15× ligation reaction buffer (57 mM Tris pH 7.5, 11 mM MgCl_2_, 11 mM DTT, and 1.1 mM ATP, supplemented with 1% Triton X-100) and incubated for 1 h at 37 °C with gentle agitation. Ligation was then performed by addition of 2000 U of T4 DNA Ligase (New England Biolabs) and incubation at 16 °C for 8 h. To reverse the crosslinking and eliminate proteins, samples were treated with 1 mg of Proteinase K and incubated at 65 °C overnight with agitation. The following day, products were treated with RNase cocktail (Ambion), to eliminate RNA, and the ligated chromatin was phenol-chloroform purified and ethanol precipitated. The quality of the 3C samples was checked as previously described^46^. In particular, the efficiency of *Bgl*II digestion was checked by PCR by amplification of a fragment across each restriction site in undigested (UND), digested not re-ligated (DIG), and ligated samples (3C) (Supplementary Fig. S2c,d).

To analyse the ICR2 *locus*, a second digestion of 3C template DNA was performed with *Bam*HI. Briefly, 8 µg of 3C DNA was digested overnight with 24 U of *Bam*HI at 37 °C. The next day, DNA was diluted in 1× T4 ligase buffer (NEB) containing 320 U of T4 ligase and incubated at 16 °C for 8 h. The ligated DNA was then phenol-chloroform purified and ethanol precipitated.

Results generated from control template, containing all possible ligation products in equal amounts, were used for normalization of 3C PCR data. One Bacterial Artificial Chromosome (BAC) (RP11-889I17) and three fosmids (G248P87866G7; G248P82515A8; G248P88924E6) covering the ICR1 region, and four BACs (RP11-1030I18; RP11-937O11; RP11-66E9; RP11-1069J4) covering the ICR2 region, were used to generate the PCR control template (Supplementary Fig. S2a,b). Equimolar amounts of each BAC and fosmid were mixed and digested with *Bgl*II, followed by ligation in a 50 µl reaction volume. The mixture was purified by phenol extraction and ethanol precipitation. An appropriate amount of DNA, which could be amplified within the linear range (2.5 ng for control template and 25 ng for 3C ligation products), was used for subsequent experiments^46,47^.

### 3C primers, PCR, and quantification

3C primers were designed with similar melting temperatures at approximately 100 bp from the *Bgl*II restriction sites in the region under study (Supplementary Table S2).

Both the 3C ligation products and control template libraries were PCR amplified (35 cycles) using GoTaq Flexi (Promega) with reagents provided by the manufacturer. PCR products were separated by 2% agarose gel electrophoresis and quantified using Image J software (https://imagej.nih.gov/ij/).

The relative interaction frequencies of pairs of fragments were calculated by dividing the amount of PCR product generated from the 3C ligation product library by the amount of PCR product obtained from the control template library^46,47^.

### 3C-SNP assay

SNPs in ligated products were analysed to define the allele specificity of chromatin associations in control lymphoblastoid cell lines (CTRL1 and CTRL2). Heterozygous SNPs, mapping within the restriction fragments generated in 3C experiments, with minor allele frequencies (MAFs) close to 0.5, as reported by UCSC (https://genome.ucsc.edu) and dbSNP (https://www.ncbi.nlm.nih.gov/projects/SNP/) databases, were selected. Analysed SNPs and their corresponding genomic positions and MAFs are reported in Supplementary Table S3.

Qualitative analysis of SNPs was performed by Sanger sequencing. In detail, 3C ligated products were separated by 2% agarose gel electrophoresis, excised, and gel purified using the MinElute Gel Extraction kit (Qiagen), according to the manufacturer’s protocol. Eluted PCR products were quantified using an ND-1000 Spectrophotometer (NanoDrop products) and sequenced using the BigDye Terminator v3.1 Cycle Sequencing Kit on an ABI 3100XL capillary sequencer (Applied Biosystems). Electropherograms were analysed using ChromasPro software 1.42 (Technelysium Pty Ltd), and aligned to the wild-type chromosome 11 NCBI reference sequence (NC_000011.10) (http://www.ncbi.nlm.nih.gov/genbank/).

Quantitative analysis of rs80047492 and rs59121562 SNP alleles and the 8F-24R (CTCF Up-ICR1) and 26R-24R (Enh A-ICR1) 3C products from CTRL1 was performed by MassARRAY analyzer 4 (Agena Bioscience), using the single base extension technique. Amplification and extension primers were designed using Assay Designer Suite v.1.0 (Agena Bioscience), and their sequences are provided in Supplementary Table S4. PCR, Shrimp Alkaline Phosphatase, and extension reactions were performed using the Complete iPLEX Pro Genotyping Reagent Set (Agena Bioscience). Amplification products were analysed using MassARRAY Typer 3.4 software (Agena Bioscience), which allows determination of SNP allele frequencies according to the different molecular weight of each allele extension product.

### 3D multicolour DNA FISH

The 3D multicolor DNA FISH assay was performed as previously described with a few modifications^48^.

To produce the two probes, we used the BAC RP11-66E9 (ICR2-Enh 2) and a mixture of the fosmids G248P87866G7, G248P82515A8 and G248P88924E6 (ICR1-CTCF Dw). 1500 ng of BAC or fosmid-mix DNA were labelled with biotin-11-dUTP (Thermo Fisher Scientific) or digoxigenin-11-dUTP (Roche Diagnostics) respectively through nick translation at 16 °C for 90 min, to obtain an average probe size of 50 bp. Probes were collected by ethanol precipitation, resuspended in Elution Solution (PhasePrep BAC DNA Kit, Sigma-Aldrich) and then quantified using a Nanodrop 1000 Spectrophotometer (Thermo Fisher Scientific). For a single experiment, 150-300 ng of each probe was precipitated with 3.5 μg of Human Cot-1 DNA (Thermo Fisher Scientific) and 20 μg of Deoxyribonucleic acid, single stranded from salmon testes (SSD, Sigma-Aldrich), and then resuspended in 6 μl of Hybridization solution (50% formamide pH 7.0 (FA)/2× saline-sodium citrate (SSC)/10% Dextran sulfate).

25 × 10^4^ lymphoblastoid cells of CTRL1, CTRL2 and BWS-ICR2 were plated on poly-lysine (Sigma-Aldrich)-treated coverslips and fixed with 3% Paraformaldehyde (PFA) in 1× PBS/0.1% TWEEN 20 (PBS-T) for 10 min at room temperature (RT). During the last minute, few drops of 0.5% Triton X-100 in 1× PBS were added and then cells were washed with 0.05% Triton X-100 in PBS three times for 3 min at RT. Cells were first permeabilised with 0.5% Triton X-100 in PBS for 10 min at RT and then were treated with RNase Cocktail Enzyme Mix (Thermo Fisher Scientific) for 1 h at 37 °C, to remove RNA. Samples were incubated overnight at RT with 20% Glycerol in PBS and then conserved at +4 °C. Cells were subjected to other steps of permeabilisation by four cycles of freeze and thaw, interleaved by soak with 20% Glycerol in PBS. Permeabilised cells were washed with 0.5% Triton X-100 in PBS for 5 min at RT and twice with 0.05% Triton X-100 in PBS for 5 min. Cells were then incubated in 0.1 M HCl for 15 min at RT, followed by a rinse with 2× SSC and then incubated in 50% FA in 2× SSC overnight at RT.

Slides were equilibrated in 2× SSC for 2 min, washed in PBS for 3 min and then treated with 0.0025% pepsin (Sigma-Aldrich) in 0.01 N HCl for 2 min at RT, to eliminate cytoskeleton. Pepsin was inactivated by two washes with 50 mM MgCl_2_ in PBS for 5 min. Nuclei were post-fixed with 1% PFA in PBS for 1 min, washed with PBS for 5 min and twice with 2× SSC, and then incubated with 50% FA in 2× SSC for at least 30 min at RT.

Hybridization between fixed cells (kept in 50% FA/2× SSC) and hybridization solution (probes in 50% FA/2× SSC/10% dextran sulphate) was performed as follows. Hybridization solution was loaded on a clean microscopic slide, coverslip with nuclei was turned upside down on the drop of hybridization mixture and sealed with rubber cement. Samples were denatured for 4 min at 75 °C and leaved to hybridise in a metallic box floating in a 37 °C water bath at least overnight. After hybridization, cells were washed three times with 2× SSC at 37 °C for 5 min and then three times with 0.1× SSC at 60 °C for 5 min, followed by a rinse with 0.2% TWEEN 20 in 4× SSC. Blocking of unspecific sites was obtained using Blocking solution (4% BSA in 4× SSC/0.2% TWEEN 20) for 30 min at 37 °C. Samples were then incubated for 35 min in a dark and wet chamber at 37 °C with the appropriate concentration of Streptavidin, Alexa Fluor 647 conjugate (Thermo Fisher Scientific) (1:1,000) or DyLight 488 Labeled Anti-Digoxigenin/Digoxin (Vector Laboratories) (1:100) diluted in Blocking solution. Samples were washed with 0.2% TWEEN 20 in 4× SSC three times for 3 min at 37 °C, equilibrated in PBS and post-fixed with 2% Formaldehyde in PBS for 10 min at RT. Finally, the 3D-fixed nuclei were washed with PBS three times for 5 min at RT, counterstained with 1 ng/μl DAPI (Thermo Fisher Scientific) in PBS for 10 min at RT and washed with PBS twice for 5 min at RT. Coverslips were mounted with Prolong Diamond (Thermo Fisher Scientific) and slides were stored at +4 °C until image acquisition. Images were acquired using Eclipse Ti-E microscope (Nikon Instruments) at 100× magnification, with an axial distance of 200 nm consecutive sections. Image analyses were performed with Volocity software (PerkinElmer). Distances (µm) between thresholded signal centroids within each nucleus were measured.

### Statistical analysis

Regarding 3C data, all experiments were performed in duplicate, from two independent 3C assays. The frequencies of associations are expressed as mean ± standard deviation. The controls mean indicates the mean of four independent 3C assays, two from CTRL1 and two from CTRL2. The mean of each pathological cell line derives from two independent 3C assays. Differences in association frequencies between controls and patients cell lines were evaluated using the two-way ANOVA test followed by the Bonferroni post-test in the GraphPad Prism program.

Regarding 3D FISH experiments, for comparisons of CTRLs and BWS-ICR2 inter-probe distances, the unpaired t-test was applied, in the GraphPad Prism program. To determine the significance between the number of CTRLs and BWS-ICR2 nuclei, classified by inter-probe distances, the Fisher’s exact test was applied.

Statistical significance is indicated as ****P ≤ 0.0001; ***P ≤ 0.001; **P ≤ 0.01; *P ≤ 0.05.

### Web Resources

dbSNP, https://www.ncbi.nlm.nih.gov/projects/SNP/.

Ensembl Genome Browser, https://www.ensembl.org/index.html.

GenBank, http://www.ncbi.nlm.nih.gov/genbank/.

ChromHMM sofware, http://compbio.mit.edu/ChromHMM/.

Image J software, https://imagej.nih.gov/ij/.

OMIM, http://www.omim.org/.

UCSC Genome Browser, https://genome.ucsc.edu.

WashU Epigenome Browser, http://epigenomegateway.wustl.edu/.

Computational and Functional Genomics/Epigenomics, http://promoter.bx.psu.edu/hi-c/virtual4c.php.

## Supplementary information

Supplementary information.

## Data Availability

The datasets generated during and/or analysed during the current study are available from the corresponding author on reasonable request.
