# Supplementary material for: Profound alterations of the chromatin architecture at chromosome 11p15.5 in cells from Beckwith-Wiedemann and Silver-Russell syndromes patients
Source: Sci Rep. 2020 May 19;10:8275. doi: 10.1038/s41598-020-65082-1 (PMC7237657; doi:10.1038/s41598-020-65082-1)
Supplement: Supplementary file 1 — Supplementary information. [file 41598_2020_65082_MOESM1_ESM.pdf]

Profound alterations of the chromatin architecture at chromosome 11p15.5 in cells from Beckwith-Wiedemann and Silver-Russell syndromes patients

Davide Rovina, Marta La Vecchia, Alice Cortesi, Laura Fontana, Matthieu Pesant, Silvia Maitz, Silvia Tabano, Beatrice Bodega, Monica Miozzo and Silvia M Sirchia

Supplementary Information

Fig. S1

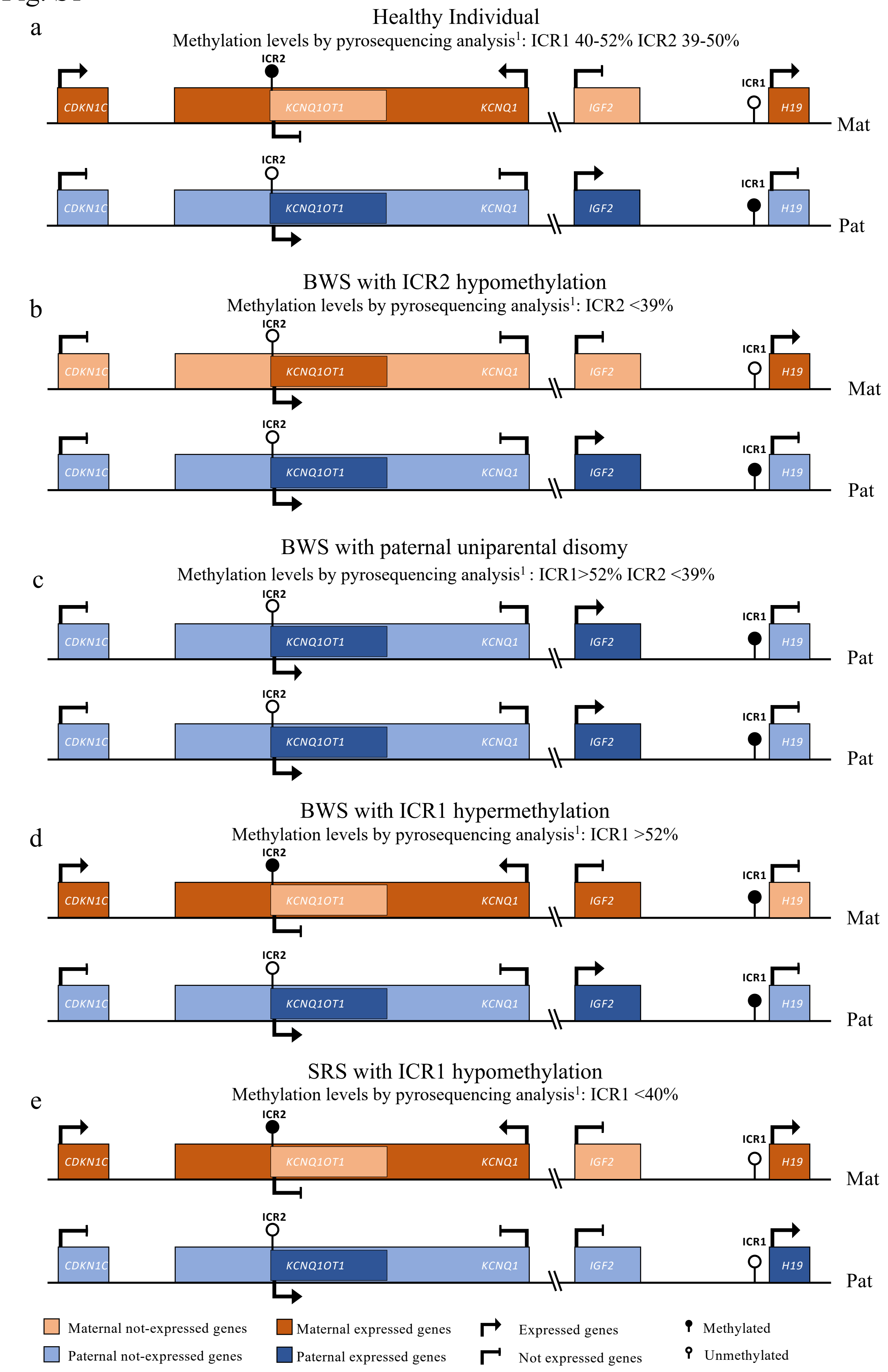
a
Healthy Individual
Methylation levels by pyrosequencing analysis[1]: ICR1 40-52% ICR2 39-50%
ICR2
ICR1
CDKN1C
KCNQ1OT1
KCNQ1
IGF2
H19
Mat
Pat
b
BWS with ICR2 hypomethylation
Methylation levels by pyrosequencing analysis[1]: ICR2 <39%
Mat
Pat
c
BWS with paternal uniparental disomy
Methylation levels by pyrosequencing analysis[1] : ICR1>52% ICR2 <39%
Pat
Pat
d
BWS with ICR1 hypermethylation
Methylation levels by pyrosequencing analysis[1]: ICR1 >52%
Mat
Pat
e
SRS with ICR1 hypomethylation
Methylation levels by pyrosequencing analysis[1]: ICR1 <40%
Mat
Pat
Maternal not-expressed genes
Maternal expressed genes
Expressed genes
Methylated
Paternal not-expressed genes
Paternal expressed genes
Not expressed genes
Unmethylated

Fig. S1. Schematic representation of the imprinted 11p15.5 region in normal and pathological conditions. The region houses two cluster of imprinted genes (*H19/IGF2* and *CDKN1C/KCNQ1OT1*) whose imprinting is regulated by ICR1 and ICR2 respectively. **(a)** In healthy individuals the maternal ICR1 allele is unmethylated whereas the paternally derived is methylated; conversely paternal ICR2 is unmethylated and the maternal one is methylated. This causes the differential expression of the genes: *IGF2* and *KCNQ1OT1* are transcribed by the paternal allele, while *H19* and *CDKN1C* are expressed by the maternal allele. Normal range of methylation by pyrosequencing analysis: ICR1 40-52%, ICR2 39-50%[1]. **(b)** BWS patients with ICR2 hypomethylation (loss of ICR2 methylation on the maternal allele, ICR2 methylation levels by pyrosequencing analysis <39%[1]) show down-regulation of *CDKN1C* and biallelic expression of *KCNQ1OT1*. The methylation of ICR1 is not affected. **(c)** in BWS individuals with paternal uniparental disomy (double paternal contribution, methylation levels by pyrosequencing analysis: ICR1>52% ICR2<39%[1]) show *IGF2* biallelically expressed. **(d)** BWS subjects with ICR1 hypermethylation (gain of ICR1 methylation on the maternal allele, ICR1 methylation levels by pyrosequencing analysis >52%[1]) display *IGF2* biallelically expressed. The methylation of ICR2 is not affected. **(e)** SRS patients with ICR1 hypomethylation (loss of ICR1 methylation on the paternal allele, ICR1 methylation levels by pyrosequencing analysis <40%[1]) show *H19* biallelically expressed and down-regulations of *IGF2*. The methylation of ICR2 is not affected.

Fig. S2

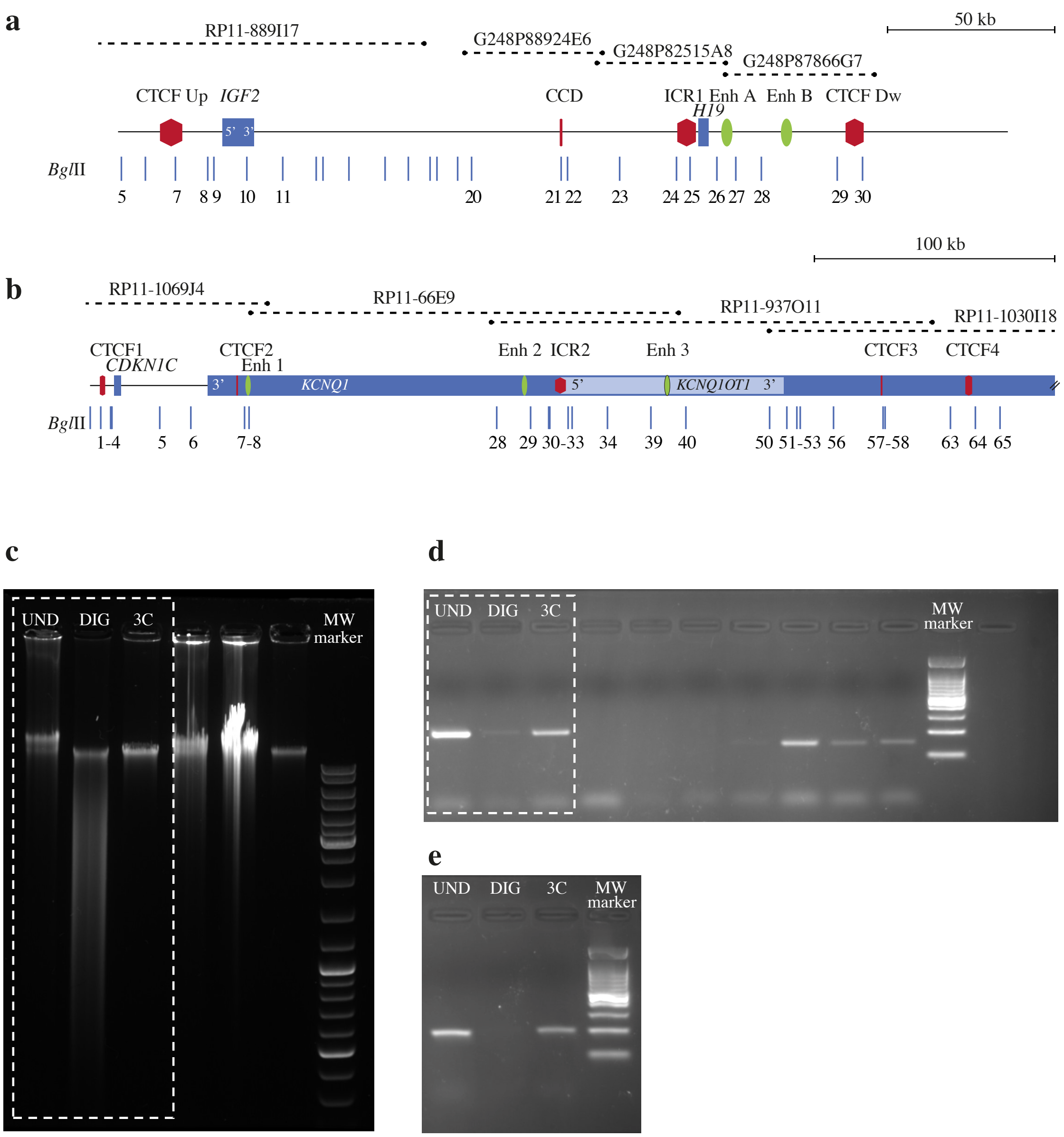

Fig. S2. Control templates for 3C PCR and quality controls of the 3C samples. (a, b) BACs and fosmids covering the ICR1 **(a)** and ICR2 **(b)** region used as control template. Each BAC and fosmid is indicated as a dashed line above the schematic representation of the corresponding domain with the indication of the *Bgl*II restriction sites. **(c, d, e)** UND, undigested cross-liked genomic DNA; DIG, digested cross-linked DNA; 3C, ligated cross-linked DNA after digestion. The samples of interest are highlighted by the white dotted rectangles on the original gel. **(c, dotted rectangle)** Analyses of the quality of 3C samples. UND DNA runs on 0.8% agarose gel at more than 10 kb. DIG DNA shows a smear-like appearance. 3C sample displays the disappearance of most of the smear-like trait. **(d, dotted rectangle and e)** Representative images of digestion efficiency on two specific restriction sites. PCR product is present in UND sample, less efficient in DIG DNA and regained in 3C sample.

Fig. S3

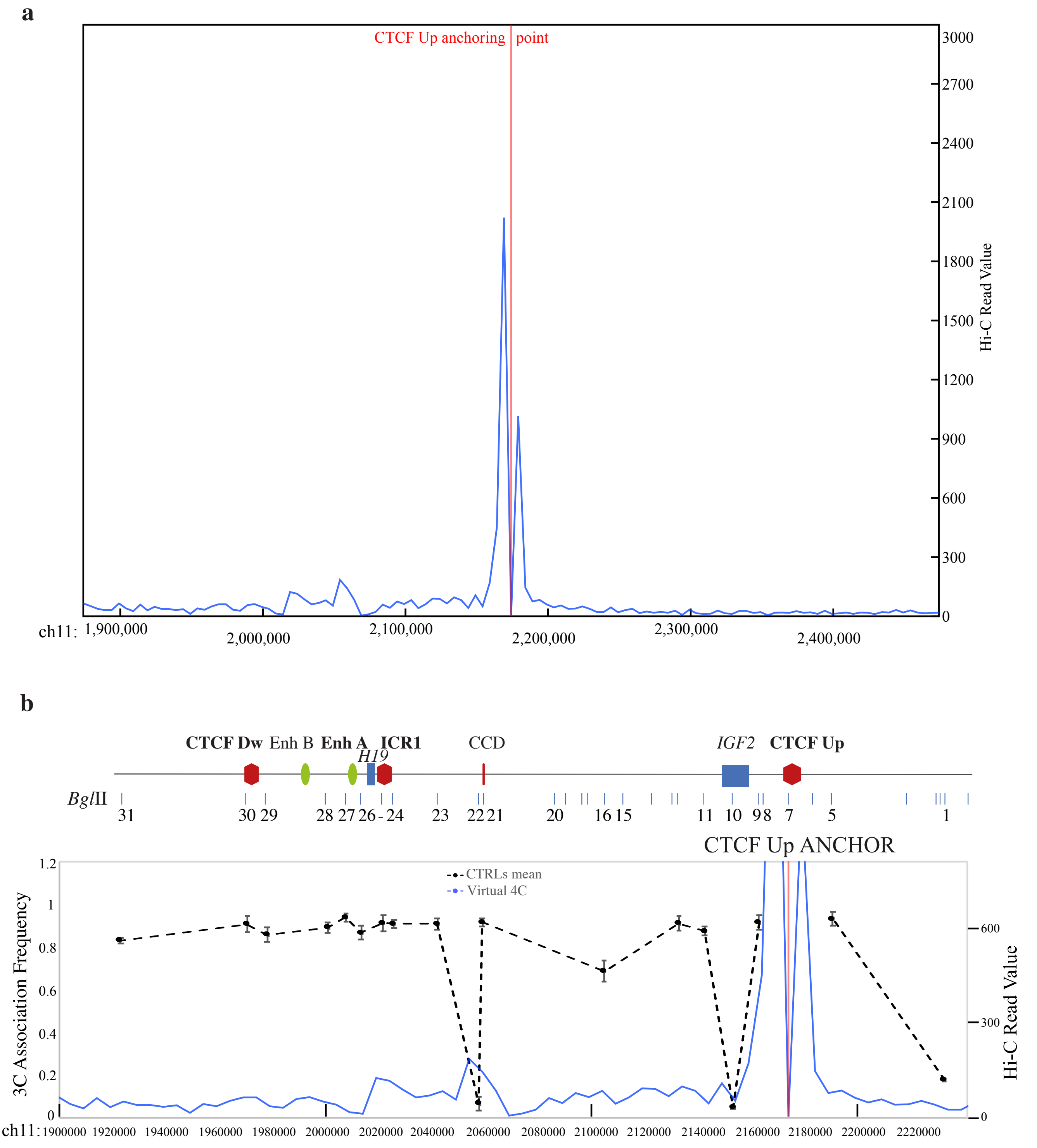
a
CTCF Up anchoring point
Hi-C Read Value
3000
2700
2400
2100
1800
1500
1200
900
600
300
0
ch11: 1,900,000
2,000,000
2,100,000
2,200,000
2,300,000
2,400,000
b
CTCF Dw
Enh B
Enh A
ICR1
H19
CCD
IGF2
CTCF Up
BglII
31
30 29
28 27 26 - 24
23
22 21
20
16 15
11
10
9 8
7
5
1
CTRLs mean
Virtual 4C
CTCF Up ANCHOR
3C Association Frequency
1.2
1
0.8
0.6
0.4
0.2
0
Hi-C Read Value
600
300
0
ch11: 1900000 1920000 1940000 1960000 1980000 2000000 2020000 2040000 2060000 2080000 2100000 2120000 2140000 2160000 2180000 2200000 2220000

c

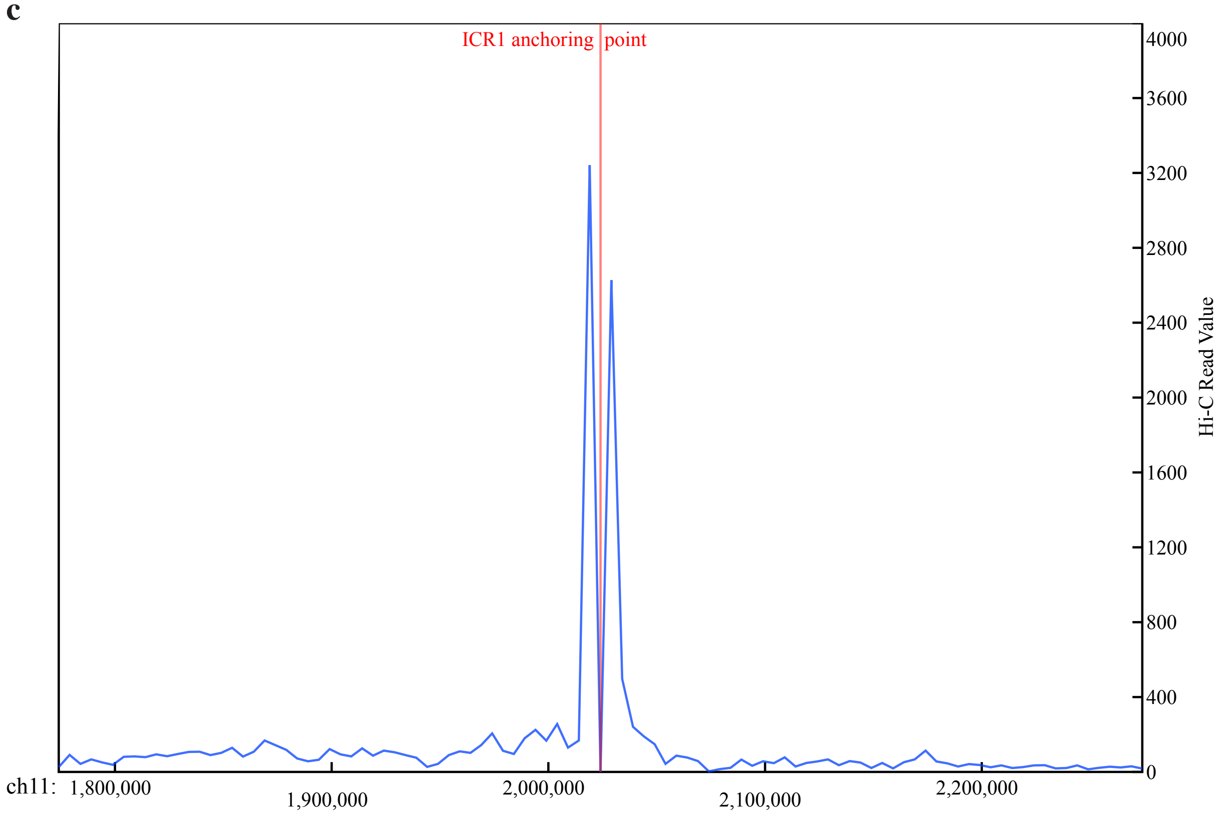
ICR1 anchoring point
4000
3600
3200
2800
2400
2000
1600
1200
800
400
0
Hi-C Read Value
ch11: 1,800,000
1,900,000
2,000,000
2,100,000
2,200,000

d

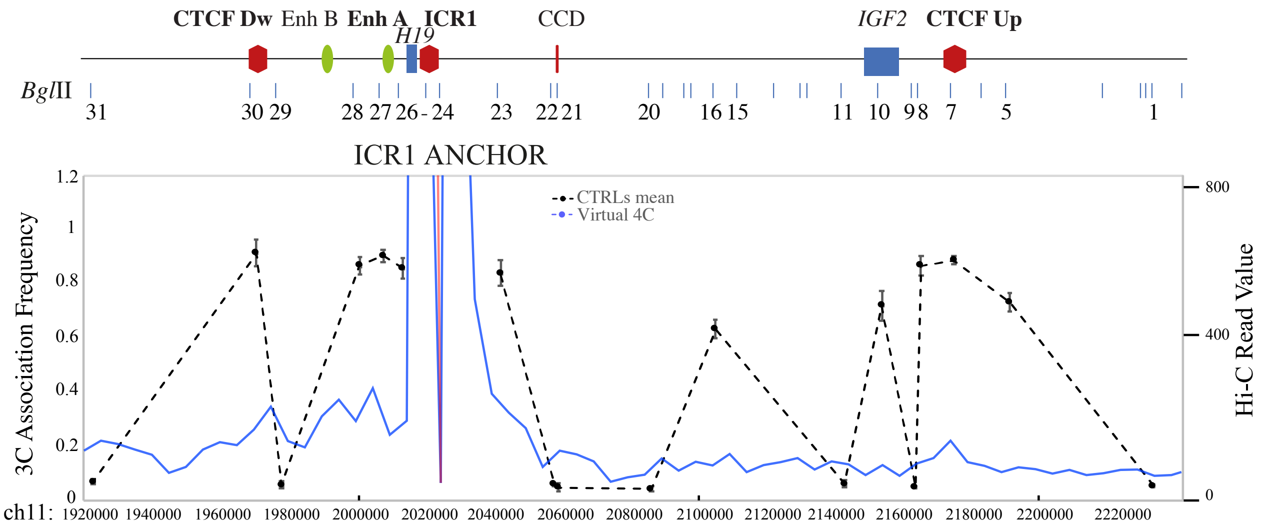
CTCF Dw
Enh B
Enh A
ICR1
H19
CCD
IGF2
CTCF Up
BglII
31
30 29
28 27 26 - 24
23
22 21
20
16 15
11
10
9 8
7
5
1
ICR1 ANCHOR
CTRLs mean
Virtual 4C
3C Association Frequency
1.2
1
0.8
0.6
0.4
0.2
0
Hi-C Read Value
800
400
0
ch11: 1920000 1940000 1960000 1980000 2000000 2020000 2040000 2060000 2080000 2100000 2120000 2140000 2160000 2180000 2200000 2220000

e

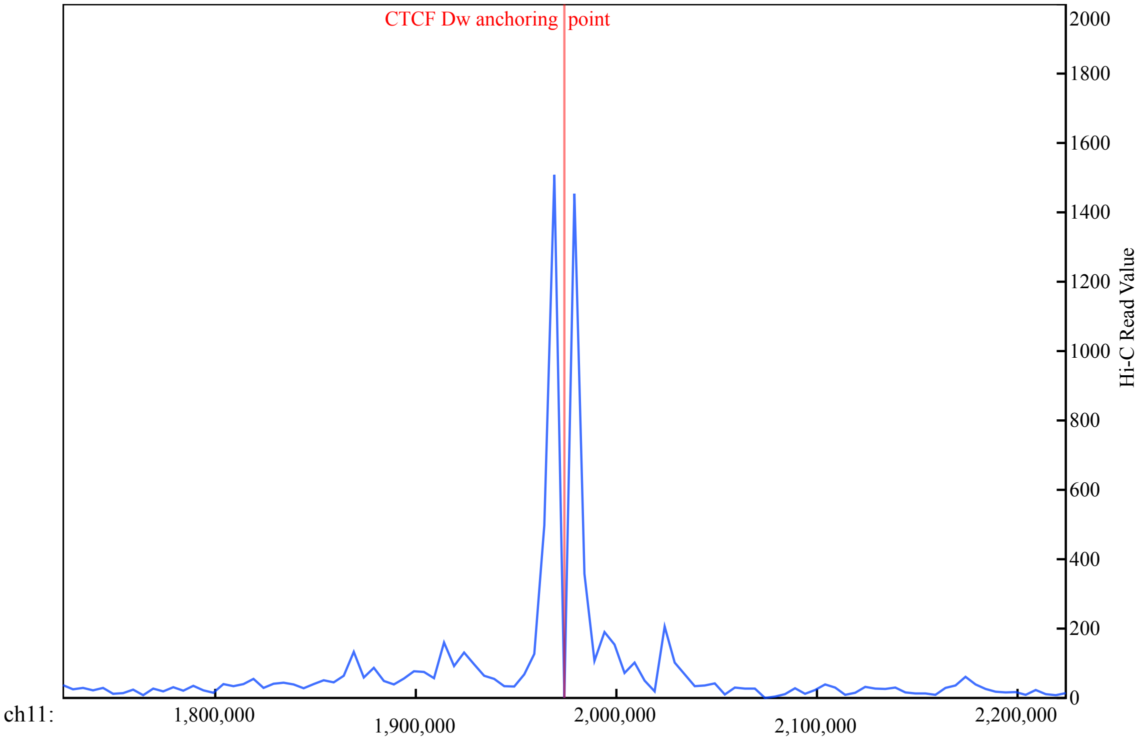

CTCF Dw anchoring point
2000
1800
1600
1400
1200
1000
800
600
400
200
0
Hi-C Read Value
ch11:
1,800,000
1,900,000
2,000,000
2,100,000
2,200,000

f

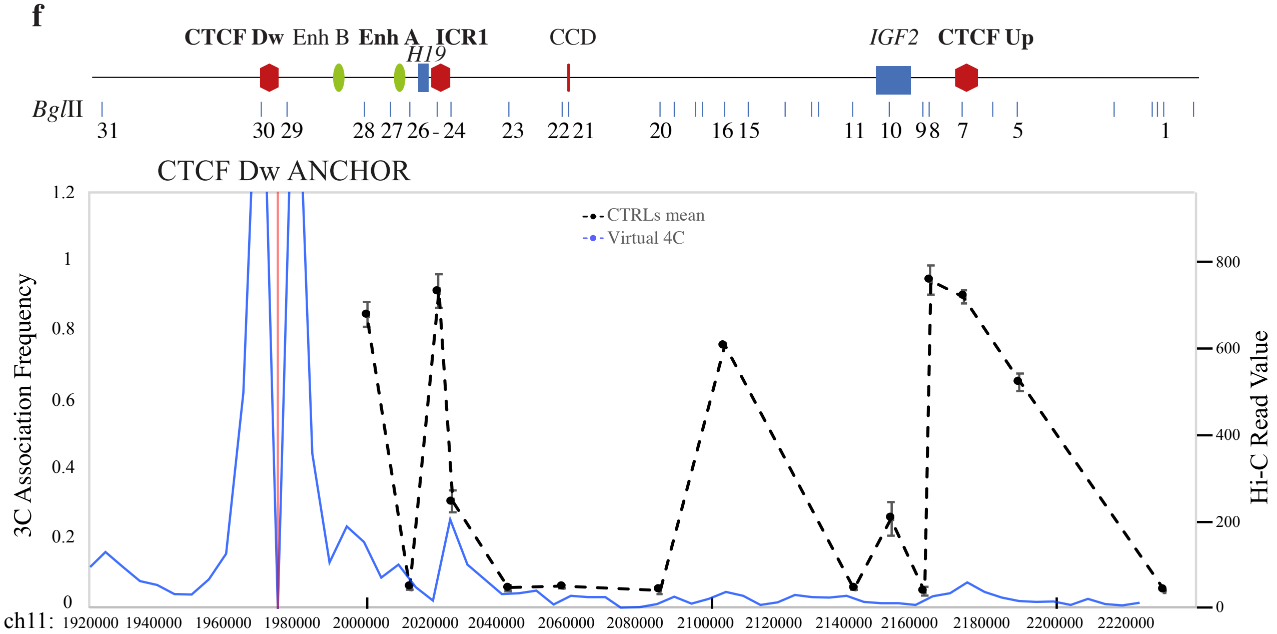

CTCF Dw
Enh B
Enh A
ICR1
H19
CCD
IGF2
CTCF Up
BglII
31
30 29
28 27 26 - 24
23
22 21
20
16 15
11
10
9 8
7
5
1
CTCF Dw ANCHOR
CTRLs mean
Virtual 4C
3C Association Frequency
Hi-C Read Value
1.2
1
0.8
0.6
0.4
0.2
0
800
600
400
200
0
ch11:
1920000 1940000 1960000 1980000 2000000 2020000 2040000 2060000 2080000 2100000 2120000 2140000 2160000 2180000 2200000 2220000

Fig. S3. Virtual 4C in *IGF2/H19 locus*.

To visualize Hi-C data obtained from the GM12878 lymphoblastoid cell line, in a virtual 4C format, we used the tool available at http://promoter.bx.psu.edu/hi-c/virtual4c.php[2] (resolution 5kb). As anchor points, we chose the same used in 3C experiments.

(a, c, e) 4C plot using CTCF Up **(a)**, ICR1 **(c)** and CTCF Down **(e)** as anchor points, the main interactions are indicated by peaks. The genomic coordinates are reported on the x-axis. **(b, d, f)** Overlay of 3C looping profile in control cell lines (dotted black) and virtual 4C plot (blue), within the same region analysed by 3C, using CTCF Up **(b)**, ICR1 **(d)** and CTCF Down **(f)** as anchors. A linear representation of the *IGF2/H19* imprinted domain with *Bgl*II restriction sites is depicted above each overlay graph. Each point in the 3C profile represents the mean ± standard deviation of 3C experiments performed in the two control cell lines (see also Fig. 2) and indicates the association frequency between the anchor and the fragment to the left of the corresponding *Bgl*II restriction site. The virtual 4C plots showed in **(b)**, **(d)** and **(f)** are a zoom of the **(a)**, **(c)** and **(e)** graphs respectively, to highlight the domain investigated in 3C experiments. The genomic coordinates are reported on the x-axis.

Fig. S4

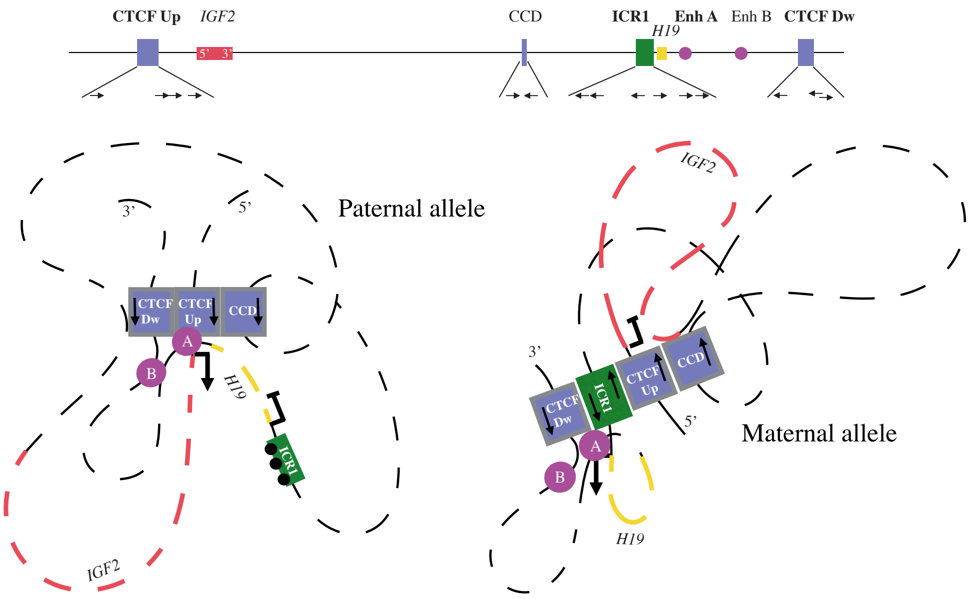

Fig. S4. Proposed models of the 3D chromatin architecture of the *IGF2/H19* domain.

A linear representation of the *IGF2/H19* imprinted domain is provided at the top. Regional elements are indicated as follows: blue rectangle, CTCF-binding site cluster; green rectangle, imprinting control region 1 (ICR1); violet circle, enhancer; red line, paternally expressed *IGF2* gene; yellow line, maternally expressed *H19* gene; black circles, CpG methylated sites; black arrows, polarity of CTCF-binding sites.

Proposed parental allele-specific chromatin conformation of the human *IGF2/H19* domain in controls is presented below, including architectural loops (involving CTCF-binding sites) and functional loops (involving regulatory elements and genes) of the paternal (left) and maternal (right) alleles. For our integrated models, we favoured interactions between the nearest convergent CTCF sites, as reported by de Wit [3] and Guo [4].

Previous published data allocated the associations CTCF Up-Enh A and CTCF Up-CTCF Dw to the paternal allele, and ICR1-CTCF Dw to the maternal allele, whereas the interaction between CTCF Up and CCD was characterised as biallelic [5–7]. We analysed our 3C data comparing cell lines from patients with pathological conditions caused by ICR1 driver defects. Since the altered allele in BWS is of maternal origin, whereas in SRS the defect is on the paternal allele, we classified interactions that were lost or significantly reduced in the BWS-ICR1 cell line, and maintained or increased in SRS-ICR1 cells (ICR1-5' IGF2; CTCF Up-3' IGF2; Fig. 4 and Fig. 5), as maternal, while we considered those that were lost or significantly decreased in SRS-ICR1 and maintained or increased in BWS-ICR1 cells (CTCF Up-CTCF Dw; CTCF Up-Enh A; Enh A-5' IGF2; CTCF Dw-5' IGF2; Fig. 4 and Fig. 5) as paternal contacts. We could verify the allele specificity of the ICR1-CTCF Up and ICR1-Enh A associations by 3C-SNP assay (see Fig. 2c). The analysis revealed that both interactions were predominantly monoallelic. Considering that the ICR1 in the paternal allele is methylated and does not bind the CTCF [8,9], we can suppose that these interactions are on the maternally-derived allele.

The methylation status of the critical CTCF-binding sites in ICR1 could change the balance of the interactions among the different CTCF clusters in the region, with the ICR1-CTCF Dw association favoured at the maternal unmethylated allele and the CTCF Up-CTCF Dw interaction favoured at the paternal methylated allele. This allele-specific chromatin structure allows the recruitment of the enhancers A and B to their target promoters in a parental allele-specific manner.

Fig. S5

a

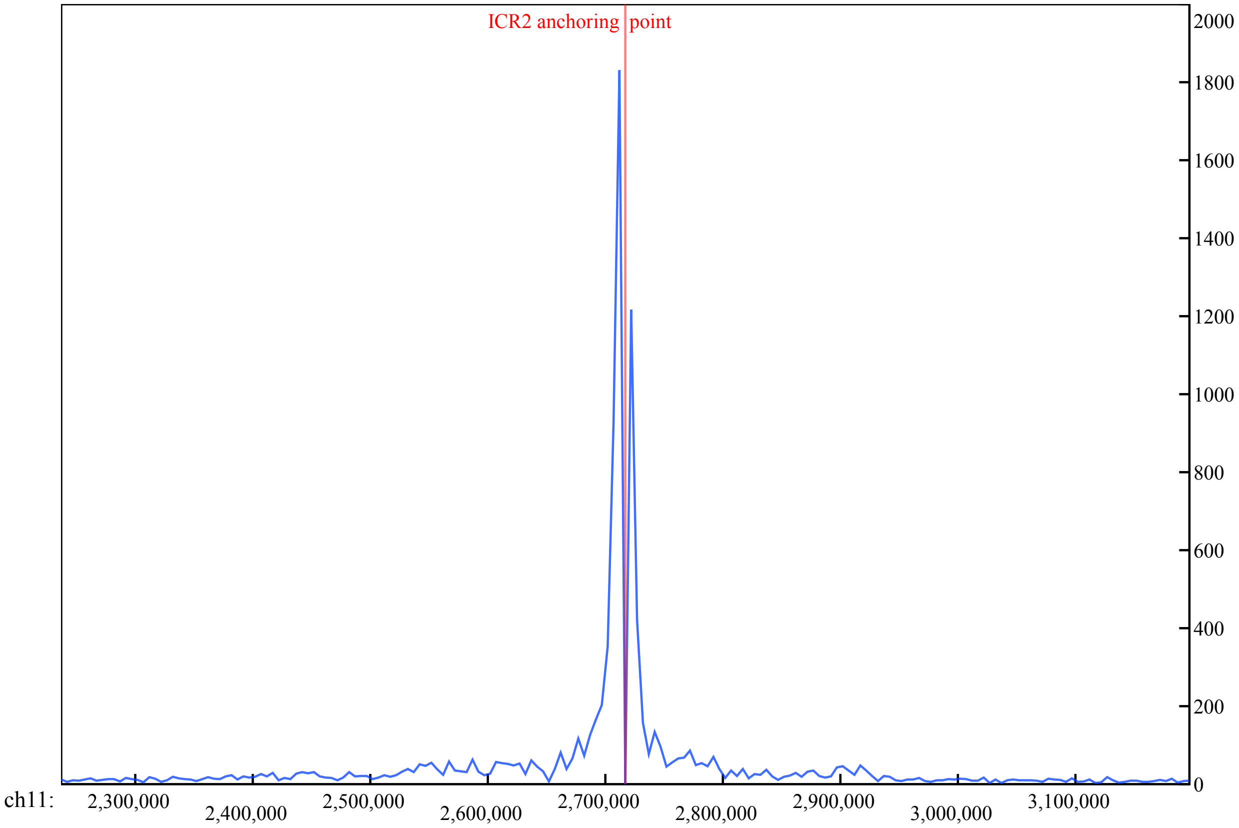
ICR2 anchoring point
2000
1800
1600
1400
1200
1000
800
600
400
200
0
Hi-C Read Value
ch11:
2,300,000
2,400,000
2,500,000
2,600,000
2,700,000
2,800,000
2,900,000
3,000,000
3,100,000

b

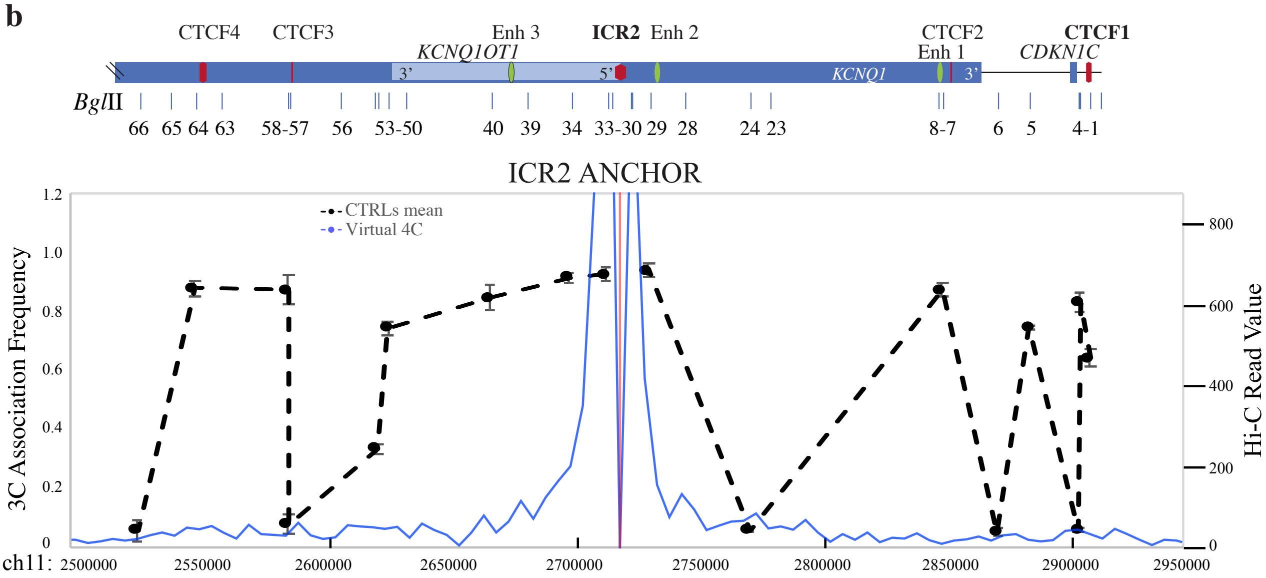
CTCF4
CTCF3
Enh 3
KCNQ1OT1
ICR2
Enh 2
CTCF2
Enh 1
CTCF1
CDKN1C
KCNQ1
BglII
66 65 64 63 58-57 56 53-50 40 39 34 33-30 29 28 24 23 8-7 6 5 4-1
ICR2 ANCHOR
CTRLs mean
Virtual 4C
3C Association Frequency
Hi-C Read Value
ch11:
2500000
2550000
2600000
2650000
2700000
2750000
2800000
2850000
2900000
2950000

c

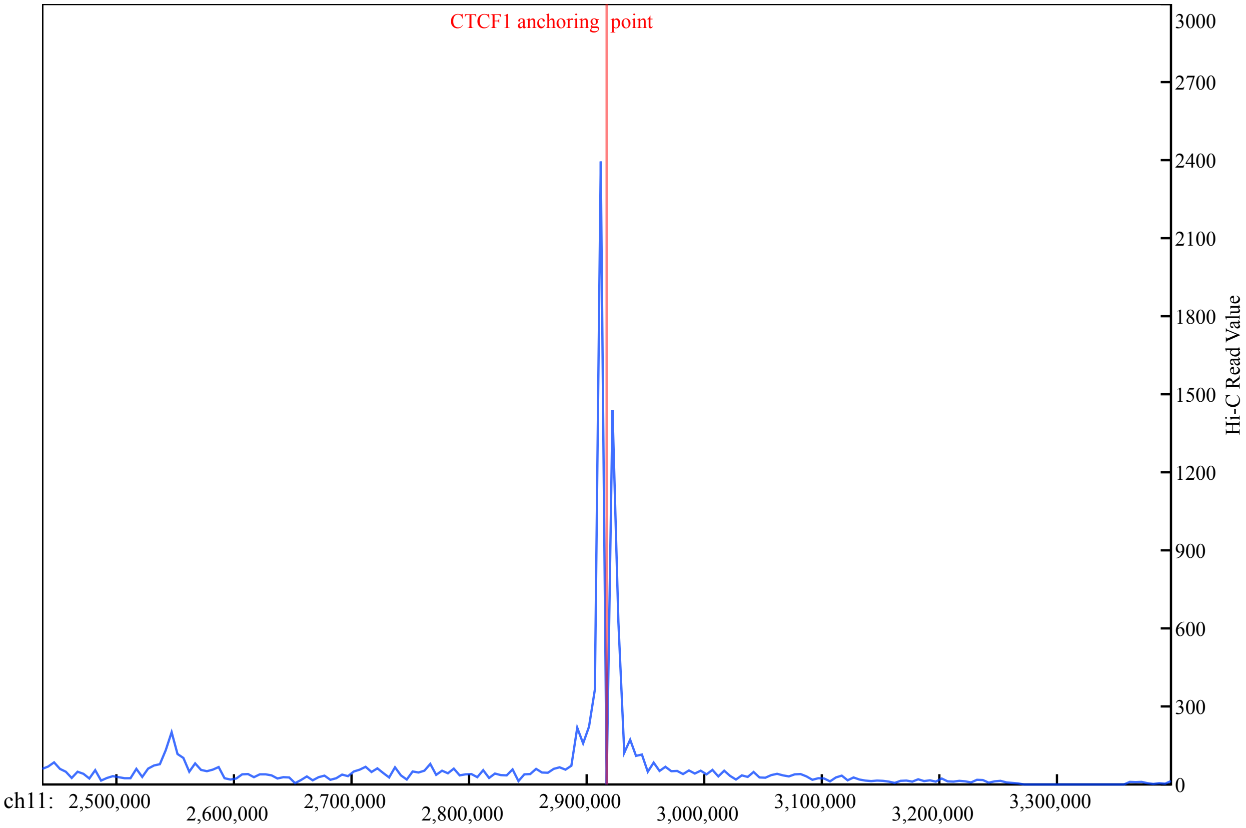
CTCF1 anchoring point
3000
2700
2400
2100
1800
1500
1200
900
600
300
0
Hi-C Read Value
ch11: 2,500,000
2,600,000
2,700,000
2,800,000
2,900,000
3,000,000
3,100,000
3,200,000
3,300,000

d

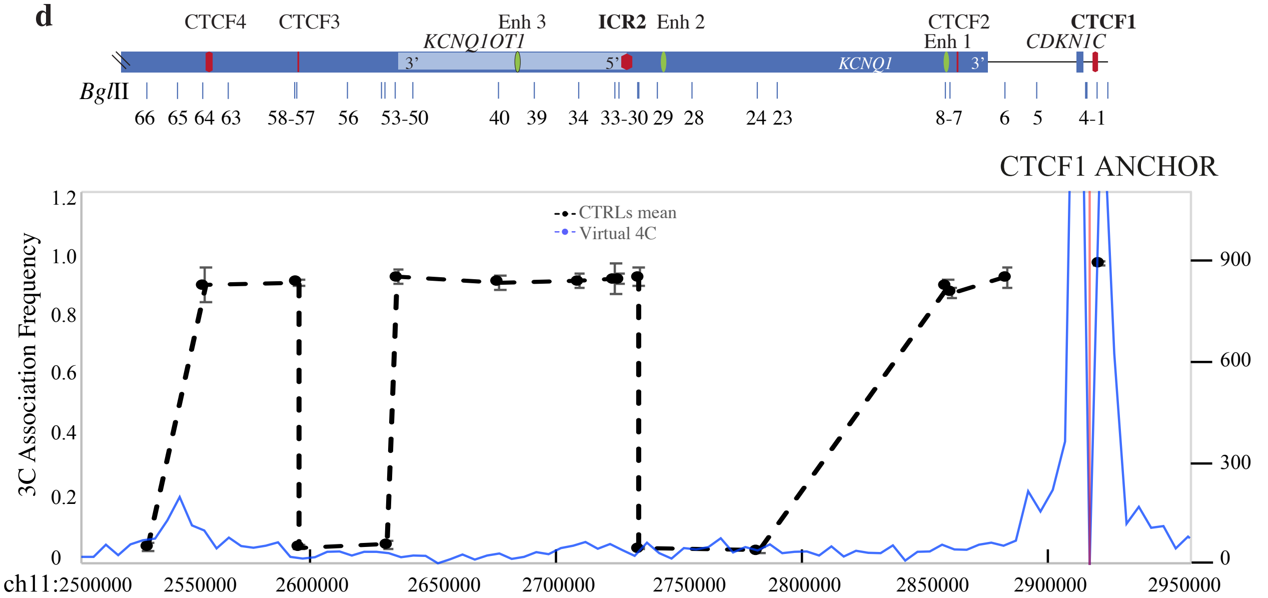
CTCF4
CTCF3
Enh 3
KCNQ1OT1
ICR2
Enh 2
CTCF2
Enh 1
CTCF1
CDKN1C
3'
5'
KCNQ1
3'
BglII
66 65 64 63 58-57 56 53-50 40 39 34 33-30 29 28 24 23 8-7 6 5 4-1
CTCF1 ANCHOR
CTRLs mean
Virtual 4C
3C Association Frequency
1.2
1.0
0.8
0.6
0.4
0.2
0
900
600
300
0
ch11:2500000
2550000
2600000
2650000
2700000
2750000
2800000
2850000
2900000
2950000

Fig. S5. Virtual 4C in *CDKN1C/KCNQ1OT1 locus*.

To visualize Hi-C data obtained from the GM12878 lymphoblastoid cell line, in a virtual 4C format, we used the tool available at http://promoter.bx.psu.edu/hi-c/virtual4c.php[2] (resolution 5kb). As anchor points, we chose the same used in 3C experiments.

(a, c) 4C plot using ICR2 **(a)** and CTCF1 **(c)** as anchor points, the main interactions are indicated by peaks. The genomic coordinates are reported on the x-axis. **(b, d)** Overlay of 3C looping profile in control cell lines (dotted black) and virtual 4C plot (blue), within the same region analysed by 3C, using ICR2 **(b)** and CTCF1 **(d)** as anchors. A linear representation of the *CDKN1C/KCNQ1OT1* imprinted domain with *Bgl*II restriction sites is depicted above each overlay graph. Each point in the 3C profile represents the mean ± standard deviation of 3C experiments performed in the two control cell lines (see also Fig. 3) and indicates the association frequency between the anchor and the fragment to the left of the corresponding *Bgl*II restriction site. The virtual 4C plots showed in **(b)** and **(d)** are a zoom of the **(a)** and **(c)** graphs respectively, to highlight the domain investigated in 3C experiments. The genomic coordinates are reported on the x-axis.

Fig. S6

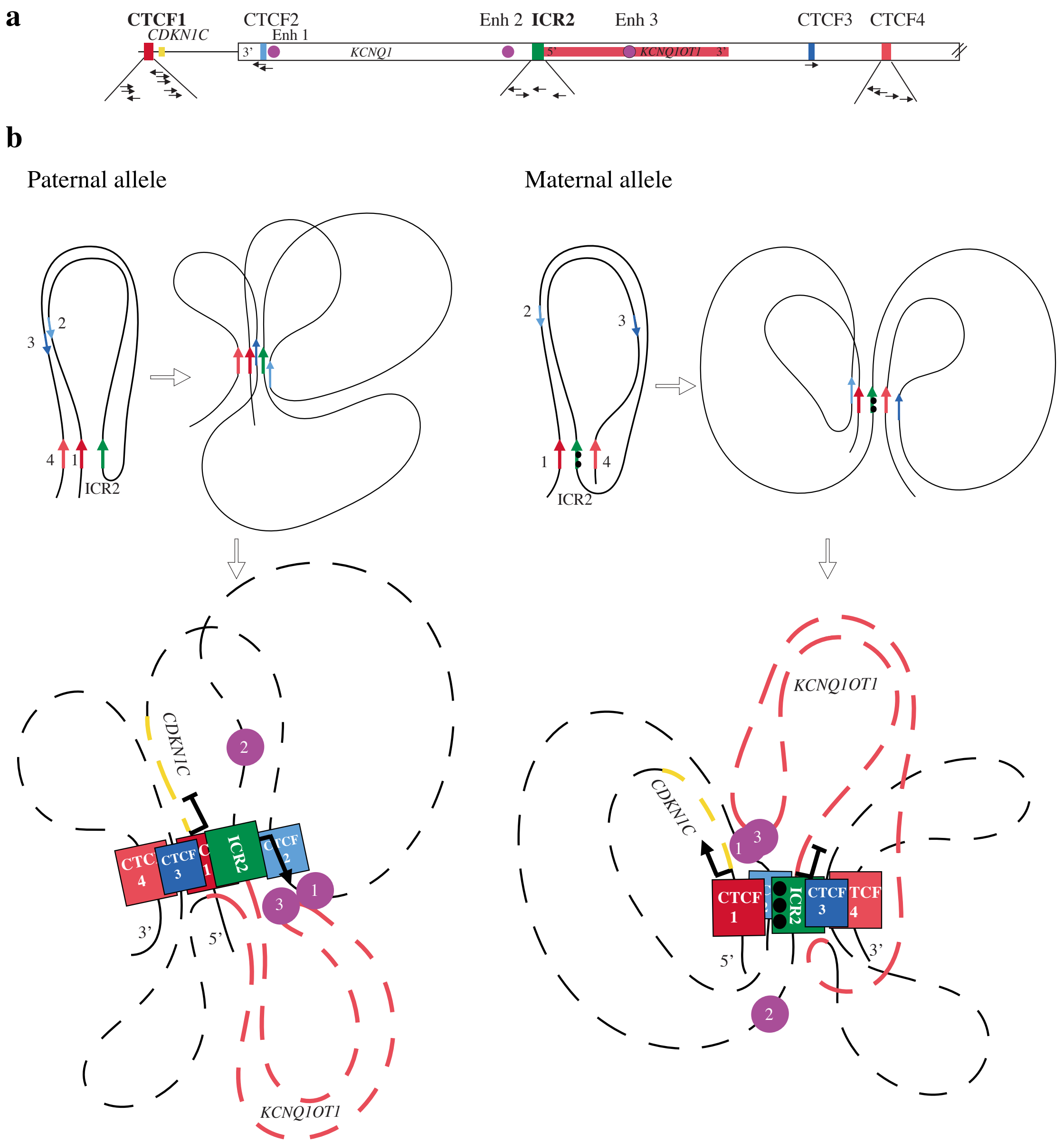
a
CTCF1
CDKN1C
CTCF2
Enh 1
KCNQ1
Enh 2
ICR2
Enh 3
KCNQ1OT1
CTCF3
CTCF4
b
Paternal allele
Maternal allele
ICR2
CDKN1C
KCNQ1OT1
CTCF 1
CTCF 3
CTCF 4
ICR2
3'
5'

Fig. S6. Proposed models of the 3D chromatin architecture of the *CDKN1C/KCNQ1OT1* domain.
(a, b) Regional elements are indicated as follows: coloured rectangles, CTCF-binding site clusters (red, CTCF1; light blue, CTCF2; blue, CTCF3; orange, CTCF4); green rectangle, imprinting control region 2 (ICR2); black circles, CpG methylated sites; violet circle, enhancer; red line, paternally expressed KCNQ1OT1 gene; yellow line, maternally expressed CDKN1C gene; arrows, polarity of the CTCF-binding sites. For our integrated models, we favoured interactions between the nearest convergent CTCF sites, as reported by de Wit [3] and Guo [4].
(a) Linear representation of the *CDKN1C/KCNQ1OT1* imprinted domain. The polarity of CTCF-binding sites is indicated by black arrows.
(b) Proposed parental allele-specific chromatin conformation of the human *CDKN1C/KCNQ1OT1* domain in controls. Simplified schematics of the possible core chromatin structure, centred on the interactions among the main CTCF-binding site clusters (CTCF1, ICR2, CTCF4), are shown above; coloured arrows indicate the polarity of the CTCF-binding site, within the CTCF cluster, that could be involved in the interaction. This core structure (CTCF hub) differs between the parental alleles. The methylation status of ICR2 may drive the orientation of ICR2 binding with CTCF1 or CTCF4. Specifically, ICR2 interacts with CTCF1 and 4 on the maternal allele (right), whereas it associates only with CTCF1 on the paternal allele (left). The CTCF4, indeed, binds the ICR2 predominantly on one allele, presumably the maternal one (see text). In the two parental models, the CTCF protein could bind the CTCF binding sites with different polarities within the ICR2, CTCF1, and CTCF4 clusters.
Below the simplified schematics are shown the chromatin conformation models of the two parental alleles displaying architectural loops (involving CTCF-binding sites) and functional loops (involving regulatory elements and genes). In our models, the two IGs alleles are oriented differently with respect to the unique plane formed by the CTCF hub; this orientation should allow the recruitment of regional enhancers to their target promoters in a parental allele-specific manner. In particular, the architectural loops bring the regional enhancers (Enh 1–3) into the proximity of 5’ KCNQ1OT1 in the paternal allele and of 5’ CDKN1C in the maternal allele. On the maternal allele, the methylation of ICR2 mapping in the KCNQ1OT1 promoter region could reinforce gene silencing. We also observed the association between the 3’ of KCNQ1OT1 and CTCF-binding sites (CTCF1 and ICR2). These loops could cooperate to the regulation of the IGs.

Fig. S7

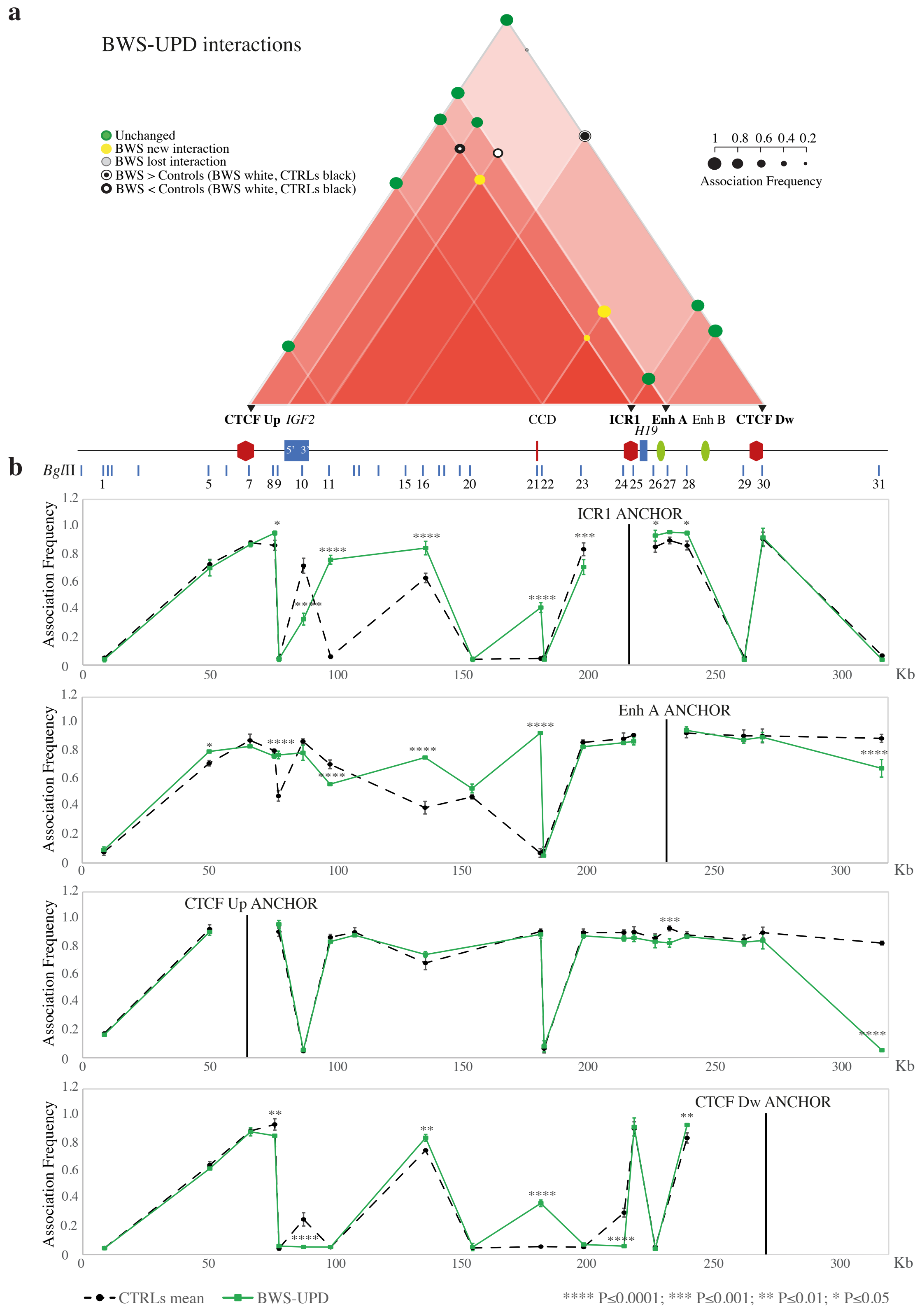
a
BWS-UPD interactions
Unchanged
BWS new interaction
BWS lost interaction
BWS > Controls (BWS white, CTRLs black)
BWS < Controls (BWS white, CTRLs black)
1 0.8 0.6 0.4 0.2
Association Frequency
CTCF Up IGF2
CCD
ICR1 Enh A Enh B CTCF Dw
H19
b
BglII
1 5 7 8 9 10 11 15 16 20 21 22 23 24 25 26 27 28 29 30 31
ICR1 ANCHOR
Enh A ANCHOR
CTCF Up ANCHOR
CTCF Dw ANCHOR
Association Frequency
Kb
CTRLs mean
BWS-UPD
**** P≤0.0001; *** P≤0.001; ** P≤0.01; * P≤0.05

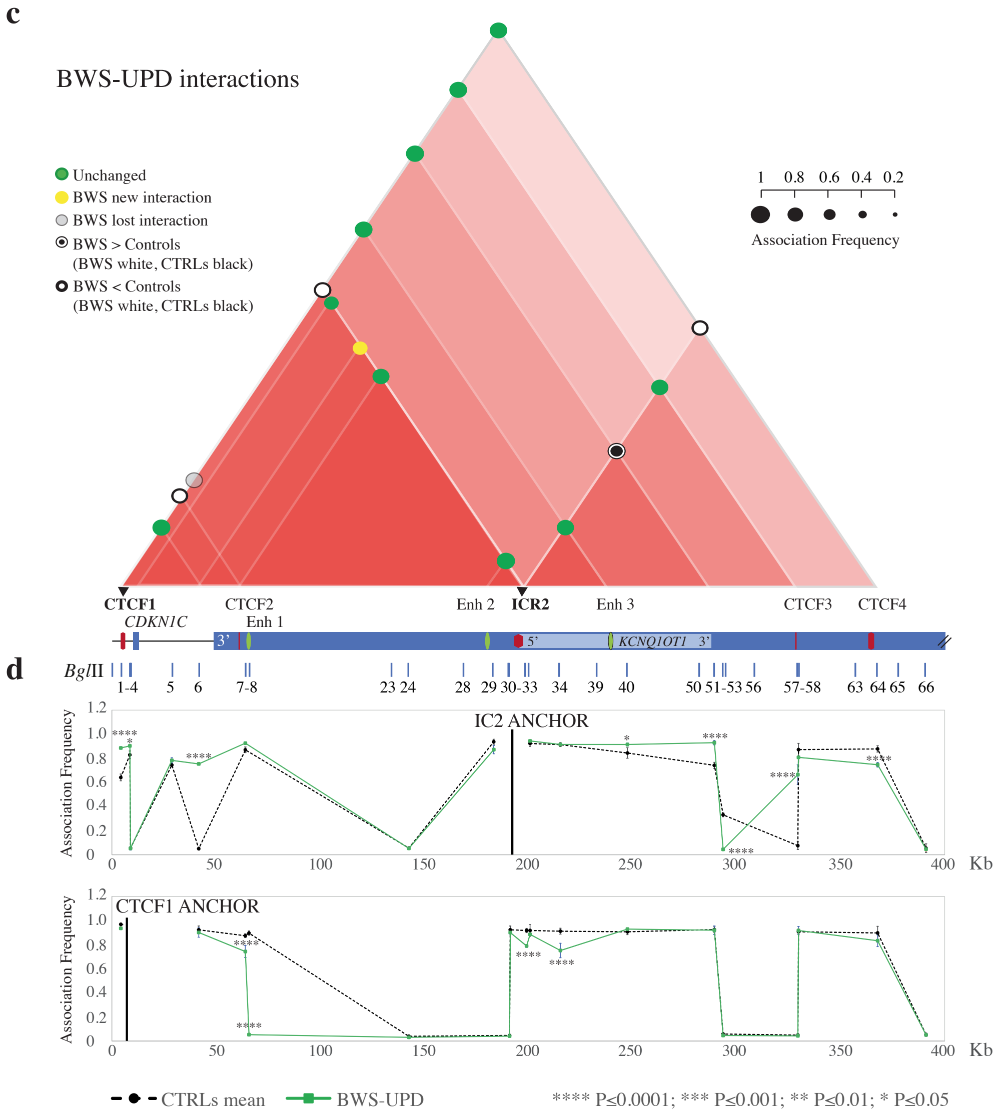

Fig. S7. Chromatin architecture alterations at the *IGF2/H19* and *CDKN1C/KCNQ1OT1* loci in BWS-UPD cells.

The entire figure is to scale. **(a and c)** Schematic showing modifications of the chromatin interactome in the ICR1 **(a)** and ICR2 **(c)** domains in the BWS-UPD cell line compared with the mean of controls. All the differences displayed were statistically significant. Red triangles, interactions between the different elements in the region. The intensity of the red colour is directly proportional to the number of interactions present in a sub-region. Coloured circles represent association frequencies: green, unchanged interaction compared with the control mean; yellow, novel interaction in the pathological cell line; light grey, interaction lost in the pathological cell line; white circle > black circle, increase of the interaction strength in the pathological cell line compared with the control mean; black circle > white circle, decrease of the interaction strength in the pathological cell line compared with the control mean. A linear representation of the *IGF2/H19* **(a)** and *CDKN1C/KCNQ1OT1* **(c)** imprinted domains is depicted below. Black triangles and bold characters indicate the anchors, used for 3C analysis.

(b and d) *IGF2/H19* **(b)** and *CDKN1C/KCNQ1OT1* **(d)** loci looping profile for the different anchors in the controls (dotted black) and BWS-UPD (green) cell lines. BglII restriction sites are indicated above. Each point in the profile represents the mean ± standard deviation of two independent 3C experiments and indicates the association frequency between the anchor and the fragment to the left of the corresponding BglII restriction site. Statistically significant differences (two-way ANOVA test) between the mean of controls and BWS-UPD are indicated by asterisks; **** P ≤ 0.0001; *** P ≤ 0.001; ** P ≤ 0.01; * P ≤ 0.05. Overall, the results represent the sum of the chromatin conformations of normal alleles (paternal and maternal) and the BWS-UPD pathological allele.

Fig. S8

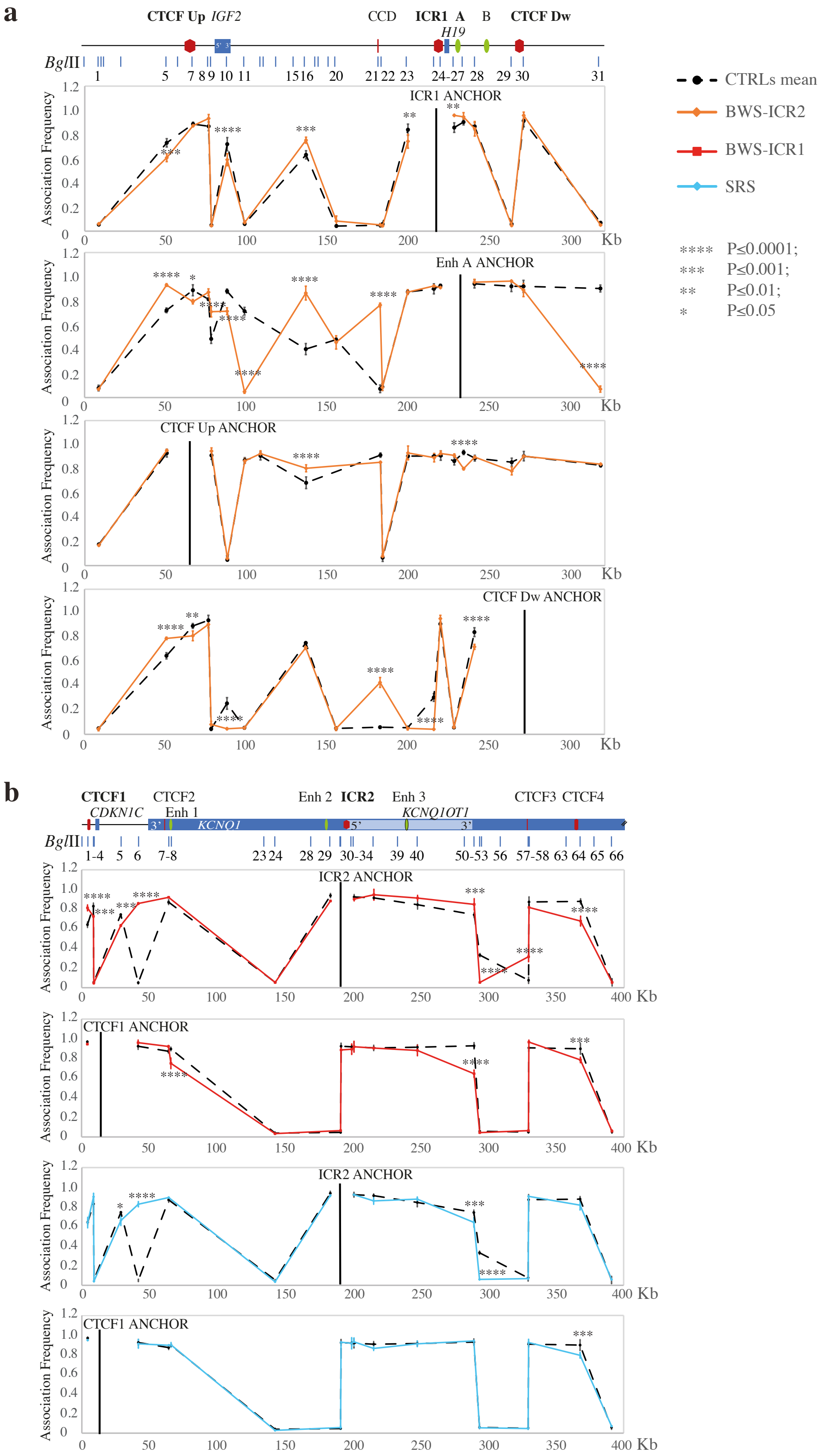
a
CTCF Up IGF2
CCD
ICR1 A B
CTCF Dw
H19
BglII
1 5 7 8 9 10 11 15 16 20 21 22 23 24-27 28 29 30 31
ICR1 ANCHOR
Enh A ANCHOR
CTCF Up ANCHOR
CTCF Dw ANCHOR
Association Frequency
Kb
CTRLs mean
BWS-ICR2
BWS-ICR1
SRS
**** P≤0.0001;
*** P≤0.001;
** P≤0.01;
* P≤0.05
b
CTCF1 CTCF2 Enh 2 ICR2 Enh 3 CTCF3 CTCF4
CDKN1C Enh 1
KCNQ1
KCNQ1OT1
1-4 5 6 7-8 23 24 28 29 30-34 39 40 50-53 56 57-58 63 64 65 66
ICR2 ANCHOR
CTCF1 ANCHOR

Fig. S8. Chromatin architecture alterations at the *IGF2/H19* or *CDKN1C/KCNQ1OT1* locus in cell lines derived from patients with driver methylation defect in the opposite locus.

(a) Looping profile alterations at the *IGF2/H19* locus in the pathological cell line with a driver methylation defect in ICR2 (BWS-ICR2, orange) compared with controls (dotted black). The schematic representation of the *IGF2/H19* domain, indicating the BglII restriction sites, is shown above.

(b) Looping profile alterations at the *CDKN1C/KCNQ1OT1* locus in the pathological cell lines with a driver methylation defect in ICR1 (BWS-ICR1, red and SRS-ICR1, light blue) compared with controls (dotted black). A schematic representation of the *CDKN1C/KCNQ1OT1* domain, indicating the BglII restriction sites, is shown above.

(a, b) Each point in the looping profile represents the mean ± standard deviation of two independent 3C experiments and indicates the association frequency between the anchor and the fragment to the left of the corresponding BglII restriction site. Statistically significant differences between the mean of the controls and pathological samples are indicated by asterisks (two-way ANOVAtest; **** $P \leq 0.0001$; *** $P \leq 0.001$; ** $P \leq 0.01$; * $P \leq 0.05$).

Fig. S9

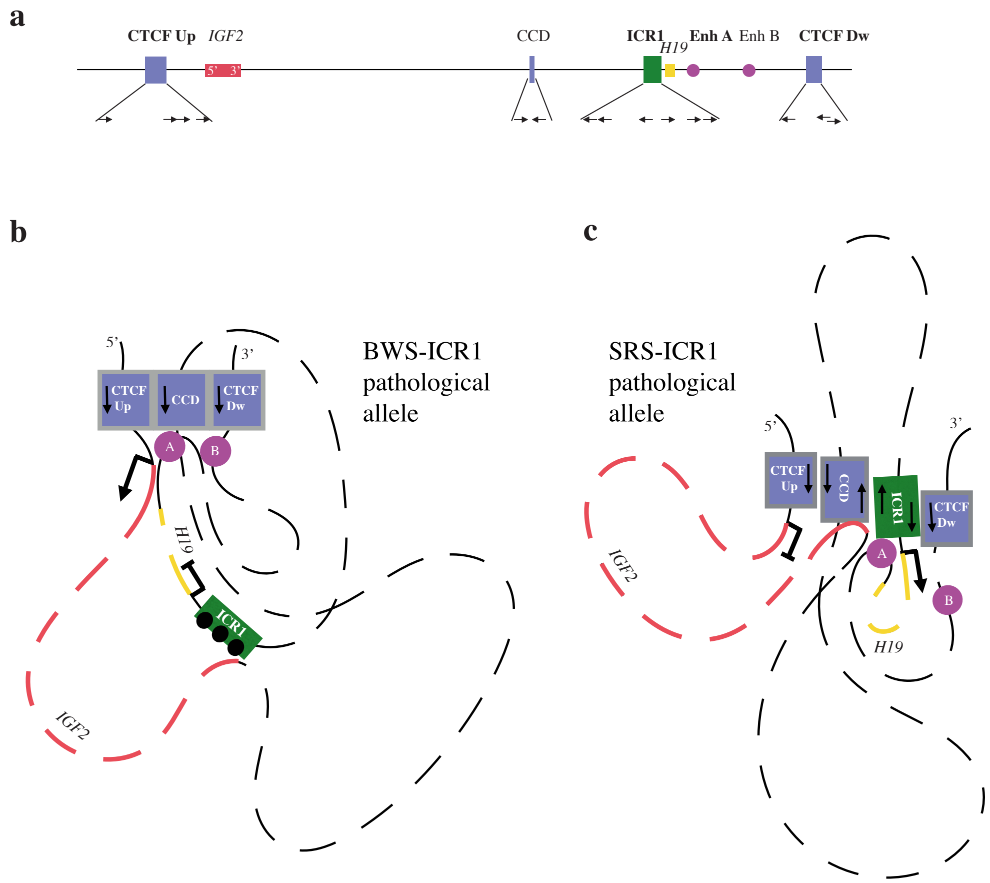

Fig. S9. Proposed simplified 3D chromatin architecture models of the *IGF2/H19* domain in BWS-ICR1 and SRS-ICR1 cells.

(a) A linear representation of the *IGF2/H19* imprinted domain is provided at the top. The polarity of CTCF-binding sites is indicated by black arrows. Regional elements are indicated as follows: blue rectangle, CTCF-binding site cluster; green rectangle, imprinting control region 1 (ICR1); violet circle, enhancer **(a** and **b)**; red line, paternally expressed IGF2 gene; yellow line, maternally expressed H19 gene; black circles, CpG methylated sites; black arrows, polarity of CTCF-binding sites. For our integrated models, we favoured interactions between the nearest convergent CTCF sites, as reported by de Wit [3] and Guo [4].

(b, c) Possible chromatin conformation of the BWS-ICR1 (maternal altered allele, **b**) and SRS-ICR1 (paternal altered allele, **c**) pathological alleles of the ICR1 domain, representing architectural and functional loops. To elaborate these models, we considered the novel contacts, compared with controls, and modifications in the interactome observed in the pathological cell lines.

In these alleles, the new chromatin conformation causes spatial repositioning of the regional enhancers, compared with the normal maternal and paternal alleles (see Fig. S4). These structures and the methylation status of ICR1 could explain the IGs expression defects observed in BWS (IGF2 upregulation and H19 downregulation) and SRS (H19 upregulation and IGF2 downregulation).

References

1. Calvello, M. *et al*. Quantitative DNA methylation analysis improves epigenotype-phenotype correlations in Beckwith-Wiedemann syndrome. *Epigenetics* **8**, 1053–1060 (2013).

2. Wang, Y. *et al*. The 3D Genome Browser: a web-based browser for visualizing 3D genome organization and long-range chromatin interactions. *Genome Biology*. **19**, 151 (2018).

3. de Wit, E. *et al.* CTCF Binding Polarity Determines Chromatin Looping. *Mol. Cell* **60**, 676–684 (2015).

4. Guo, Y. *et al.* CRISPR Inversion of CTCF Sites Alters Genome Topology and Enhancer/Promoter Function. *Cell* **162**, 900–910 (2015).

5. Nativio, R. *et al.* Cohesin is required for higher-order chromatin conformation at the imprinted IGF2-H19 locus. *PLoS Genet.* **5**, e1000739 (2009).

6. Nativio, R. *et al.* Disruption of genomic neighbourhood at the imprinted IGF2-H19 locus in Beckwith-Wiedemann syndrome and Silver-Russell syndrome. *Hum. Mol. Genet.* **20**, 1363–1374 (2011).

7. Ito, Y., Nativio, R. & Murrell, A. Induced DNA demethylation can reshape chromatin topology at the IGF2-H19 locus. *Nucleic Acids Res.* **41**, 5290–5302 (2013).

8. Bell, A. C. & Felsenfeld, G. Methylation of a CTCF-dependent boundary controls imprinted expression of the Igf2 gene. *Nature* **405**, 482–485 (2000).

9. Hark, A. T. *et al.* CTCF mediates methylation-sensitive enhancer-blocking activity at the H19/Igf2 locus. *Nature* **405**, 486–489 (2000).

Table S1. CTCF-binding sites, genomic position, orientation and references.

Region	CTCF Cluster	Genomic Position (GRCh37/hg19)	Orientation	References
IGF2/H19	CTCF Down	Chr11:1,970,198-1,970,547	Reverse	ENCODE; Nativio et al. 2009; Nativio et al. 2011; Ito et a. 2013; Guo et al. 2015; Sparago et al. 2018
		Chr11:1,974,896-1,975,015	Forward	
		Chr11:1,975,515-1,975,864	Forward	
	ICR1	Chr11:2,019,539-2,019,888	Reverse	
		Chr11:2,020,102-2,020,451	Reverse	
		Chr11:2,021,019-2,021,368	Reverse	
		Chr11:2,021,873-2,022,222	Forward	
		Chr11:2,022,345-2,022,694	Forward	
		Chr11:2,023,318-2,023,667	-	
		Chr11:2,024,121-2,024,470	Reverse	
	CCD	Chr11:2,058,228-2,058,351	Forward	
		Chr11:2,059,092-2,059,441	Reverse	
	CTCF UP	Chr11:2,177,705-2,178,054	Reverse	
		Chr11:2,173,345-2,173,434	Reverse	
		Chr11:2,172,676-2,173,025	Reverse	
		Chr11:2,171,453-2,171,802	Reverse	
CDKN1C/KCNQ1OT1	CTCF4	Chr11:2,548,313-2,548,662	Reverse	ENCODE; Guo et al. 2015
		Chr11:2,550,898-2,551,247	Reverse	
		Chr11:2,552,703-2,552,855	Forward	
		Chr11:2,554,040-2,554,280	Forward	
	CTCF3	Chr11:2,587,789-2,587,830	Reverse	

	ICR2	Chr11:2,713,601-2,713,950	Forward	
		Chr11:2,715,068-2,715,511	Forward	
		Chr11:2,719,640-2,720,069	Reverse	
		Chr11:2,720,929-2,721,278	Forward	
	CTCF2	Chr11:2,858,292-2,858,333	Forward	
		Chr11:2,858,397-2,858,438	Forward	
	CTCF1	Chr11:2,909,705-2,909,908	Reverse	
		Chr11:2,912,404-2,912,871	Forward	
		Chr11:2,913,459-2,913,562	Reverse	
		Chr11:2,914,077-2,914,426	Forward	
		Chr11:2,914,978-2,915,327	-	
		Chr11:2,916,403-2,916,826	Reverse	

Table S2. Primers used for the 3C PCRs

Primers used for the 3C PCRs of the ICR1 locus

Anchor Primer		ICR1-locus Primers		Analysed elements
ICR1-24R	AGCCTGGATGATAAGAGCGA	1F	GAGGGCTGGTGAGAGTGG	
		5F	CGGAAGTCTTGTAGGGGAGG	
		7F	CTGGGGAAGCGAGTTAAGAAC	CTCF Up
		8F	AGGAATTCTGAATCCCCTTTCTT	
		9F	GACACGGCTGCGGACTTG	
		10F	GCCAGAGTGAGGAAGGAGTT	5' *IGF2*
		11F	ACAACTCTAGGAAGTGGCACA	3' *IGF2*
		15R	TGCTAGAGTGAATAGATGGGTGT	
		20F	ACTTTTACGCTGTTGGTGGG	
		21F	CCCTTCCTACCAGCCATTGT	CCD
		22F	CTCTCTGTCGGCATCCTCC	
		23F	ACAGAAATGGGCAATTCTTCCC	
		25R	CATGACACTGAAGCCCTCG	
		26R	GCTATGCCTAGTGTGGTTACC	Enh A
		27R	CCCTCTCTGCATGTCCGTG	
		28R	AGGTGGAGGAACAAAGGCAG	
		29R	TGAGGAGTCTTTGCAGCTCT	CTCF Dw
		30R	GAGAGTGGGACTGAGGGAAC	

Anchor Primer		ICR1-locus Primers		Analysed elements
CTCF Up-7F	CTGGGGAAGCGAGTTAAGAAC	1F	GAGGGCTGGTGAGAGTGG	
		5F	CGGAAGTCTTGTAGGGGAGG	
		8R	TTGGAAGGCTGGGAGACAAT	
		9R	GCTAGAGGCACTTTACCGC	5’ *IGF2*
		10R	CCCGAGGACTCCACATTTCT	3’ *IGF2*
		11R	CACAGGCTAAAACCATGGACA	
		15R	TGCTAGAGTGAATAGATGGGTGT	
		20R	CGTGCTTGTGTCTTTATGATAGG	CCD
		21R	ATAAGGGAAAGGAGGCAGGG	
		22R	AGGCAAGGGAAAGGAGAGAC	
		23R	CCTGGTCTCCTGAACCATCC	
		24R	AGCCTGGATGATAAGAGCGA	ICR1
		25R	CATGACACTGAAGCCCTCG	
		26R	GCTATGCCTAGTGTGGTTACC	Enh A
		27R	CCCTCTCTGCATGTCCGTG	
		28R	AGGTGGAGGAACAAAGGCAG	
		29R	TGAGGAGTCTTTGCAGCTCT	CTCF Dw
		30R	GAGAGTGGGACTGAGGGAAC	

Anchor Primer		ICR1-locus Primers		Analysed elements
Enh A-26R	GCTATGCCTAGTGTGGTTACC	1F	GAGGGCTGGTGAGAGTGG	
		5F	CGGAAGTCTTGTAGGGGAGG	
		7F	CTGGGGAAGCGAGTTAAGAAC	CTCF Up
		8F	AGGAATTCTGAATCCCCTTTCTT	
		9F	GACACGGCTGCGGACTTG	
		10F	GCCAGAGTGAGGAAGGAGTT	5’ *IGF2*
		11F	ACAACTCTAGGAAGTGGCACA	3’ *IGF2*
		15R	TGCTAGAGTGAATAGATGGGTGT	
		20F	ACTTTTACGCTGTTGGTGGG	
		21F	CCCTTCCTACCAGCCATTGT	CCD
		22F	CTCTCTGTCGGCATCCTCC	
		23F	ACAGAAATGGGCAATTCTTCCC	
		24F	CCAAAGTTAGTCCAGCCTGC	
		25F	TTCGCCCGTGGAAACGTC	ICR1
		27R	CCCTCTCTGCATGTCCGTG	
		28R	AGGTGGAGGAACAAAGGCAG	
		29R	TGAGGAGTCTTTGCAGCTCT	CTCF Dw
		30R	GAGAGTGGGACTGAGGGAAC	

Anchor Primer		ICR1-locus Primers		Analysed elements
CTCF Dw-30F	TGTGGGCTTGAGATGAGAGG	1F	GAGGGCTGGTGAGAGTGG	
		5F	CGGAAGTCTTGTAGGGGAGG	
		7F	CTGGGGAAGCGAGTTAAGAAC	CTCF Up
		8F	AGGAATTCTGAATCCCCTTTCTT	
		9F	GACACGGCTGCGGACTTG	
		10F	GCCAGAGTGAGGAAGGAGTT	5’ *IGF2*
		11F	ACAACTCTAGGAAGTGGCACA	3’ *IGF2*
		15R	TGCTAGAGTGAATAGATGGGTGT	
		20F	ACTTTTACGCTGTTGGTGGG	
		21F	CCCTTCCTACCAGCCATTGT	CCD
		22F	ACAGAAATGGGCAATTCTTCCC	
		24F	CCAAAGTTAGTCCAGCCTGC	
		25F	TTCGCCCGTGGAAACGTC	ICR1
		26F	TGTGTGATGCCTGACAAGC	
		28F	GGACAGCCATCATCTCCTCG	

Primers used for the 3C PCRs of the ICR2 locus

Anchor Primer		ICR2-locus Primers		Analysed elements
3F	CTCAAAGGCAGGCTGGTTG	1F	GCTTGGAGAGGCTTTGAGG	CTCF1
		5R	AGGAGCCTCGTTTCTCAGTG	
		6R	AGAGGGCAGGAATGAACTGA	CTCF2
		7R	ACACAAGAAGCTCTCCAGGT	Enh 1
		23R	TCCTCTCGACCTACCCTCAA	
		29R	TTAGACTCACCAGCCATCCC	
		30R	TCAGCATCCAACAGGGGTAG	
		31R	CAGGCTCTGGGTCCTTTTG	ICR2
		32R	CTAATGCCAAGTTGCCCTGT	
		33R	CTTGGGGCAGGAGGGATAC	
		39R	GGCACCCAAGTCCATTCTG	Enh 3
		50R	TGCTTTTCTCACCTAACAACATC	
		51R	CAAAACTGGACCAGCACAAA	
		56R	TGGCTCAGCAGGTGACAG	
		57R	GGACGGCTCTTCTTCTCGTA	CTCF3
		63R	ATGATGTCCCGTCGGCAG	CTCF4
		65R	GGAGTTCTGTGTGGACCCAG	

Anchor Primer		ICR2-locus Primers		Analysed elements
ICR2-31R	CAGGCTCTGGGTCCTTTTG	1F	GCTTGGAGAGGCTTTGAGG	CTCF1
		3F	CTCAAAGGCAGGCTGGTTG	CTCF1
		4F	GGGAGGGGCATGGTCCTT	
		5F	GGTAGGTGTGGTGACTGAGG	*CDKN1C*
		6F	CAGTGCTTTGTGGCTTCTTG	
		7F	GGAGGGGACACTATGGTTGT	CTCF2
		23R	TCCTCTCGACCTACCCTCAA	
		29F	AGTGTCTAACATTTGGGCCG	Enh 2
		32R	CTAATGCCAAGTTGCCCTGT	
		33R	CTTGGGGCAGGAGGGATAC	
		39R	GGCACCCAAGTCCATTCTG	Enh 3
		50R	TGCTTTTCTCACCTAACAACATC	
		51R	CAAAACTGGACCAGCACAAA	
		56R	TGGCTCAGCAGGTGACAG	
		57R	GGACGGCTCTTCTTCTCGTA	CTCF3
		63R	ATGATGTCCCGTCGGCAG	CTCF4
		65R	GGAGTTCTGTGTGGACCCAG	

Primers used for the 3C PCRs between the ICR1 locus and the ICR2 locus

Anchor Primer		ICR2-locus Primers		Analysed elements
ICR1-24R	AGCCTGGATGATAAGAGCGA	1F	GCTTGGAGAGGCTTTGAGG	CTCF1
		3F	CTCAAAGGCAGGCTGGTTG	CTCF1
		5F	AGAGGGCAGGAATGAACTGA	*CDKN1C*
		6F	ACACAAGAAGCTCTCCAGGT	
		7F	GGAGGGGACACTATGGTTGT	CTCF2
		29F	AGTGTCTAACATTTGGGCCG	
		30F	CCAGAGCCACAGAGTTGTCA	
		31F	CCCTGCTGCCTCTCTTCAG	
		32F	CAGCAATCAGAATAGCAGCAAA	ICR2
		33F	ACCAACGAATGAATGTGCCT	
		39F	AGTCCAAAGAACAAAACACCCT	
		50F	TGCCATCTAGAAACCACCTACA	
		51F	TGACTGAATGTTTCCCCGTTA	
		56F	GCTACTCACCACCACAGACT	
		57F	GAGAGCCCCATCCACTACAG	
		63F	TTATGGGGTCTTTCTGGGGC	
		64F	ATGAGACGGATACCACTGCC	CTCF4
		65F	AGCAGGACCAAGGCTGTC	

Anchor Primer		ICR1-locus Primers		Analysed elements
ICR2-31R	CAGGCTCTGGGTCCTTTTG	5R	TCAGGGTCGAACTTGGTGAC	
		7R	GCCACAGGATAGGTCTGGAA	
		8R	TTGGAAGGCTGGGAGACAAT	
		9R	GCTAGAGGCACTTTACCGC	5' *IGF2*
		10R	CCCGAGGACTCCACATTTCT	3' *IGF2*
		11R	CACAGGCTAAAACCATGGACA	
		20R	CGTGCTTGTGTCTTTATGATAGG	CCD
		22R	AGGCAAGGGAAAGGAGAGAC	
		23R	CCTGGTCTCCTGAACCATCC	
		24R	AGCCTGGATGATAAGAGCGA	ICR1
		25R	CATGACACTGAAGCCCTCG	
		26R	GCTATGCCTAGTGTGGTTACC	Enh A
		27R	CCCTCTCTGCATGTCCGTG	
		28R	AGGTGGAGGAACAAAGGCAG	
		29R	TGAGGAGTCTTTGCAGCTCT	CTCF Dw
		30R	GAGAGTGGGACTGAGGGAAC	

Table S3. SNPs analysed for allele specificity of chromatin associations with relative genomic position and Minor Allele Frequency (MAF).

SNP	Type	Genomic position	MAF (European)
rs80047492	A/T	NC_000011.10:g.2003802	A:0.47<br>T:0.53
rs59121562	A/T	NC_000011.10:g.2003804	A:0.47<br>T:0.53
rs2283196	C/A	NC_000011.10:g.2704522	C:0.63<br>A:0.37
rs2283197	C/G/T	NC_000011.10:g.2704549	C:0.54<br>G:0.34<br>T:0.12

Table S4. PCR and extension primers used for quantitative evaluation of SNPs rs80047492 and rs59121562 in genomic DNA and 8-24 and 26-24 3C products of the CTRL1.

Primer	Sequence
PCR primer Fw	5'-acgttggatgTCCCCCTTTTGAGAAGTCAC -3'
PCR primer Rev	5'-acgttggatgTAAGAGCGAAACTCTGTCTC -3'
Extension primer rs80047492	5'-GAAACTCTGTCTCAAAAAAAAA-3'
Extension primer rs59121562	5'-TTGTCATAATTTTCTGTTATATAT-3'
